# HEMNMA-3D: Cryo Electron Tomography Method Based on Normal Mode Analysis to Study Continuous Conformational Variability of Macromolecular Complexes

**DOI:** 10.3389/fmolb.2021.663121

**Published:** 2021-05-19

**Authors:** Mohamad Harastani, Mikhail Eltsov, Amélie Leforestier, Slavica Jonic

**Affiliations:** ^1^IMPMC-UMR 7590 CNRS, Sorbonne Université, Muséum National d'Histoire Naturelle, Paris, France; ^2^Department of Integrated Structural Biology, Institute of Genetics and Molecular and Cellular Biology, Illkirch, France; ^3^Laboratoire de Physique des Solides, UMR 8502 CNRS, Université Paris-Saclay, Paris, France

**Keywords:** cryo electron tomography, continuous conformational changes, flexible-reference alignment, normal mode analysis, nucleosomes *in situ*

## Abstract

Cryogenic electron tomography (cryo-ET) allows structural determination of biomolecules in their native environment (*in situ*). Its potential of providing information on the dynamics of macromolecular complexes in cells is still largely unexploited, due to the challenges of the data analysis. The crowded cell environment and continuous conformational changes of complexes make difficult disentangling the data heterogeneity. We present HEMNMA-3D, which is, to the best of our knowledge, the first method for analyzing cryo electron subtomograms in terms of continuous conformational changes of complexes. HEMNMA-3D uses a combination of elastic and rigid-body 3D-to-3D iterative alignments of a flexible 3D reference (atomic structure or electron microscopy density map) to match the conformation, orientation, and position of the complex in each subtomogram. The elastic matching combines molecular mechanics simulation (Normal Mode Analysis of the 3D reference) and experimental, subtomogram data analysis. The rigid-body alignment includes compensation for the missing wedge, due to the limited tilt angle of cryo-ET. The conformational parameters (amplitudes of normal modes) of the complexes in subtomograms obtained through the alignment are processed to visualize the distribution of conformations in a space of lower dimension (typically, 2D or 3D) referred to as space of conformations. This allows a visually interpretable insight into the dynamics of the complexes, by calculating 3D averages of subtomograms with similar conformations from selected (densest) regions and by recording movies of the 3D reference's displacement along selected trajectories through the densest regions. We describe HEMNMA-3D and show its validation using synthetic datasets. We apply HEMNMA-3D to an experimental dataset describing *in situ* nucleosome conformational variability. HEMNMA-3D software is available freely (open-source) as part of ContinuousFlex plugin of Scipion V3.0 (http://scipion.i2pc.es).

## 1. Introduction

Cryogenic electron microscopy (cryo-EM) image collection and analysis technique referred to as single-particle analysis (SPA) allows near-atomic structural resolution of purified biomolecular complexes (*in vitro*). It is based on the principle of reconstructing a three dimensional (3D) structure from two dimensional (2D) parallel-beam projection images of vitrified specimens containing many copies of the same macromolecular complex at unknown orientations and positions. The 3D reconstruction requires extracting macromolecular complexes (particles) from the collected images into individual (single-particle) images and determining the particle orientation and position in every single-particle image. On the other hand, cryogenic electron tomography (cryo-ET) is gaining popularity for studying biomolecular complexes in their native environments (*in situ*). Cryo-ET requires the acquisition of multiple 2D projection images of the specimen in a range of orientations. In most practices, the specimen is physically rotated around a single axis (perpendicular to the electron beam) inside the cryo electron microscope. An image is collected at each tilting angle in a specific range (e.g., −60 to 60° with a step of 1°), yielding a tilt series representing 2D projections of the specimen. The tilt series is then used to computationally reconstruct a 3D volume called tomogram. A tomographic reconstruction typically contains hundreds of copies of a target biomolecular complex at unknown orientations and positions. These copies are then identified and extracted into individual (single-particle) volumes called subtomograms, either manually or semi-automatically (via template matching methods). Subtomograms suffer from a low signal-to-noise ratio (SNR), which is due to exposing the sample to a low electron dose during data acquisition in order to preserve the fragile biological structure. Additionally, subtomograms suffer from the so-called missing wedge artifacts, which are due to inability to include in the 3D reconstruction the images from all orientations (the maximum tilt angle in the microscope is usually limited to ± 60°). The missing wedge artifacts are often observed as elongation along the beam axis, blurring, and distracting caustics in the subtomograms. Due to the low SNR and the missing wedge artifacts, cryo-ET data processing is mainly based on rigid-body aligning and averaging many subtomograms to enhance the data quality and reveal the targeted biomolecular structure (Leigh et al., 2019).

The primary technique for macromolecular structural determination is so-called subtomogram averaging (StA), in which subtomograms are classified, rigid-body aligned and averaged into 3D density maps iteratively (Mahamid et al., 2016; Schur et al., 2016; Wan and Briggs, 2016; Albert et al., 2017; Böck et al., 2017; Bykov et al., 2017; Pfeffer et al., 2017; Riedel et al., 2017; Wan et al., 2017; Davies et al., 2018; Guo et al., 2018; Hutchings et al., 2018; Kovtun et al., 2018; Mosalaganti et al., 2018; Park et al., 2018; Kaplan et al., 2019; Rapisarda et al., 2019). However, with recent instrumentation and software development, more research moves in the direction of studying single-particle subtomograms individually (with no or a minimum of averaging) by developing new methods for denoising, missing wedge correction, and 3D reconstruction (Zhang and Ren, 2012; Moebel and Kervrann, 2020; Zhai et al., 2020).

Biomolecular complexes are not rigid but flexible entities with gradual (continuous) conformational transitions, and this flexibility is usually referred to as continuous conformational variability. If not properly taken into account, conformational heterogeneity limits the resolution of the resulting 3D structure. However, SPA research in the last decade has shown that disentangling the different conformations and identifying the conformational transitions from heterogeneous samples is valuable to study molecular mechanisms of action of complexes (Dashti et al., 2014; Jin et al., 2014; Zhou et al., 2015; Abeyrathne et al., 2016; Banerjee et al., 2016; Haselbach et al., 2018). The majority of available SPA computational methods rely on optimized biochemical specimen preparation protocols and data classification, and simplify the problem of conformational heterogeneity by assuming that the data can be classified into a small number of different conformations (Penczek et al., 2006, 2011; Fu et al., 2007; Elad et al., 2008; Scheres, 2012; Lyumkis et al., 2013). However, some SPA methods explicitly take into account continuous conformational variability and aim at determining the full conformational distribution (Dashti et al., 2014; Jin et al., 2014; Sorzano et al., 2014; Katsevich et al., 2015; Tagare et al., 2015; Frank and Ourmazd, 2016; Andén and Singer, 2018; Harastani et al., 2020). They represent images in a low-dimensional space, referred to as space of conformations or energy landscape, and allow a 3D visualization of conformational changes along trajectories in this space. For more information on SPA methods for continuous conformational variability analysis, the reader is referred to the recent reviews by Jonić (2017) and Sorzano et al. (2019).

Methods reported to deal with cryo-ET data heterogeneity are based on rigid-body alignment and can be classified into (i) post-alignment classification approaches, and (ii) simultaneous alignment and classification approaches (Förster et al., 2008; Scheres et al., 2009; Stölken et al., 2011; Xu et al., 2012; Chen et al., 2014; Bharat and Scheres, 2016; Himes and Zhang, 2018). In the first family, the starting point is usually the covariance matrix representing the similarities of each pair of aligned subtomograms. The covariance matrix serves as a basis for a classification technique with some variants including dimensionality reduction. The second family of methods is based on competitive alignment. An example of the competitive alignment is a multireference alignment in which a subtomogram is compared with a set of different references provided by an expert user based on a prior knowledge and, then, attributed to the reference that yields the highest similarity score. Another example is maximum-likelihood-based alignment, where each subtomogram contributes to all references with a probability. The main drawback of the post-alignment classification approaches is that the classification is heavily dependent on the alignment quality that degenerates with broadly heterogeneous specimens. The main drawback of the simultaneous alignment and classification methods is that the number of classes must be decided and set prior to the use of the methods. Besides, the methods that require prior knowledge of the specimen's anticipated conformations are prone to overfitting and data misinterpretation. Finally, as macromolecular complexes are not rigid but flexible entities with continuous conformational transitions, particles assigned to the same class will rarely, if ever, have perfectly identical conformations. For more information regarding the classification based techniques for cryo-ET conformational heterogeneity, the reader is referred to a recent review on the available techniques by Castaño-D́ıez and Zanetti (2019).

**Figure 1:**
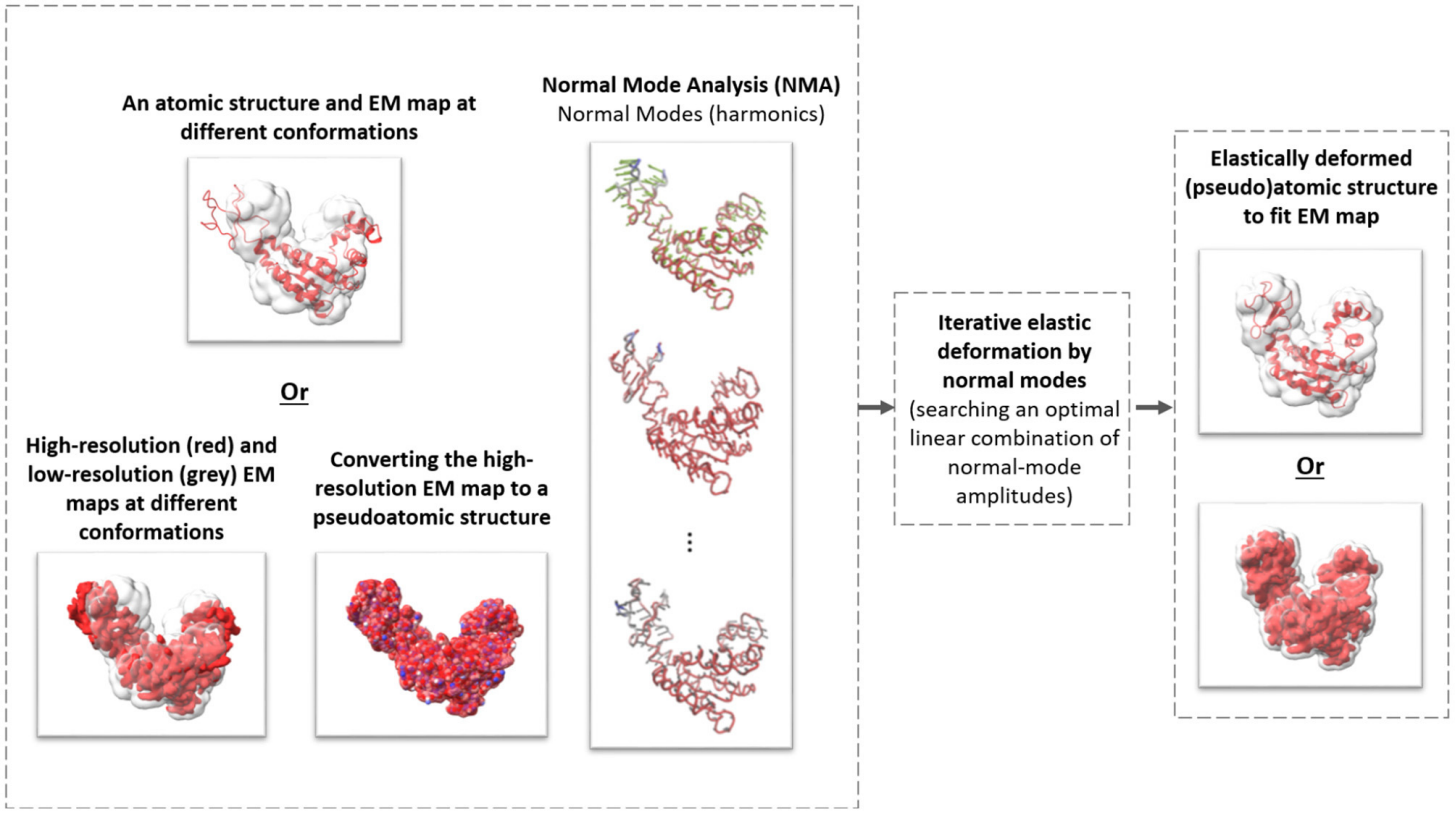
The general scheme of elastic deforming of a reference structure (atomic or pseudoatomic) using normal modes to fit a density map (e.g., an EM map or a subtomogram average).

Existing multivariate statistical analysis techniques adapted to cryo-ET analysis of continuous flexibility of particular systems have been used previously (e.g., Mattei et al., 2016). However, to the best of our knowledge, no method is currently available that has been specifically designed for cryo-ET analysis of continuous conformational variability of a general-case macromolecular complex. In this article, we present one such method, named HEMNMA-3D, which allows analyzing continuous conformational variability of macromolecular complexes by cryo-ET. It is inspired by HEMNMA, a method for continuous conformational variability analysis in SPA (Jin et al., 2014; Sorzano et al., 2014; Harastani et al., 2020). HEMNMA interprets the conformation in each cryo-EM single-particle image by comparing this image with 2D projections of a 3D reference (an atomic structure or a density map) deformed elastically using normal modes. Normal Mode Analysis (NMA) is a method for molecular mechanics simulation. One of its main applications is elastic deformation of an existing atomic structure of one conformation to fit an electron microscopy density map (EM map) of a different conformation of the same macromolecule, which is usually known as normal mode flexible fitting and allows obtaining atomic resolution models for the EM map (Tama et al., 2004a,b). However, as HEMNMA, HEMNMA-3D can use normal modes of an atomic structure or a density map. As in the case of the reference density-map structure in HEMNMA, HEMNMA-3D converts the density map into a collection of 3D Gaussian functions, referred to as pseudoatoms, and computes normal modes of this pseudoatomic structure, following the procedures described in Nogales-Cadenas et al. (2013), Jin et al. (2014), and Jonić and Sorzano (2016). HEMNMA-3D uses the atomic or pseudoatomic normal modes to elastically deform the 3D reference to match the conformation of the complex in the given series of subtomograms (3D data) (see Figure 1). More precisely, HEMNMA-3D uses a combination of elastic (based on normal modes) and rigid-body 3D-to-3D iterative alignments of the 3D reference to match the conformation, orientation, and position of the complex in each subtomogram, and includes compensation for the missing wedge. The conformational parameters (amplitudes of normal modes) of the complexes in subtomograms obtained through the alignment are then processed to visualize the distribution of conformations in a space of lower dimension (typically, 2D or 3D) referred to as space of conformations. This space allows a visually interpretable insight into the dynamics of complexes, by calculating 3D averages of subtomograms with similar conformations from selected (densest) regions and by recording movies of the 3D reference's displacement along selected trajectories in the densest regions.

In this article, we describe HEMNMA-3D and show its validation using synthetic datasets. Additionally, we show an application of HEMNMA-3D with an experimentally obtained dataset for *in situ* nucleosome conformational variability. HEMNMA-3D software is available freely (open-source) as part of ContinuousFlex plugin of Scipion V3.0 (http://scipion.i2pc.es). The article is organized as follows: section 2 describes building blocks of HEMNMA-3D workflow, notably, in 2.5 we describe the software developed for this method. In section 3, we present (i) the process of synthesis of test datasets and HEMNMA-3D validation using these synthetic test data, and (ii) use of HEMNMA-3D with experimental, *in situ* nucleosome data, and we discuss these results. The conclusions are provided in section 4.

## 2. Materials and Methods

**Figure 2:**
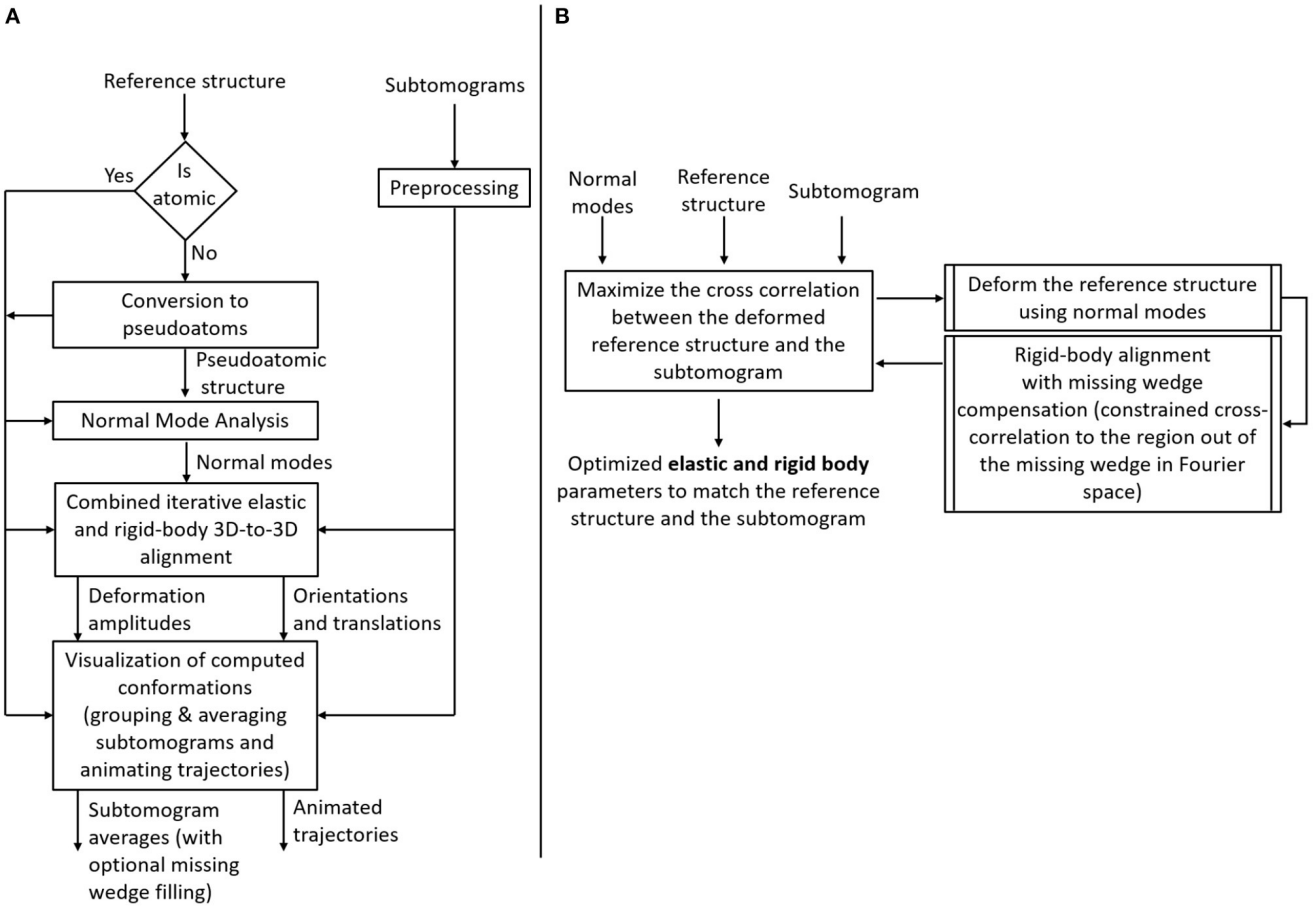
Flowchart of HEMNMA-3D. **(A)** Workflow. **(B)** Combined iterative elastic and rigid-body 3D-to-3D alignment step (the core module of HEMNMA-3D).

**Figure 3:**
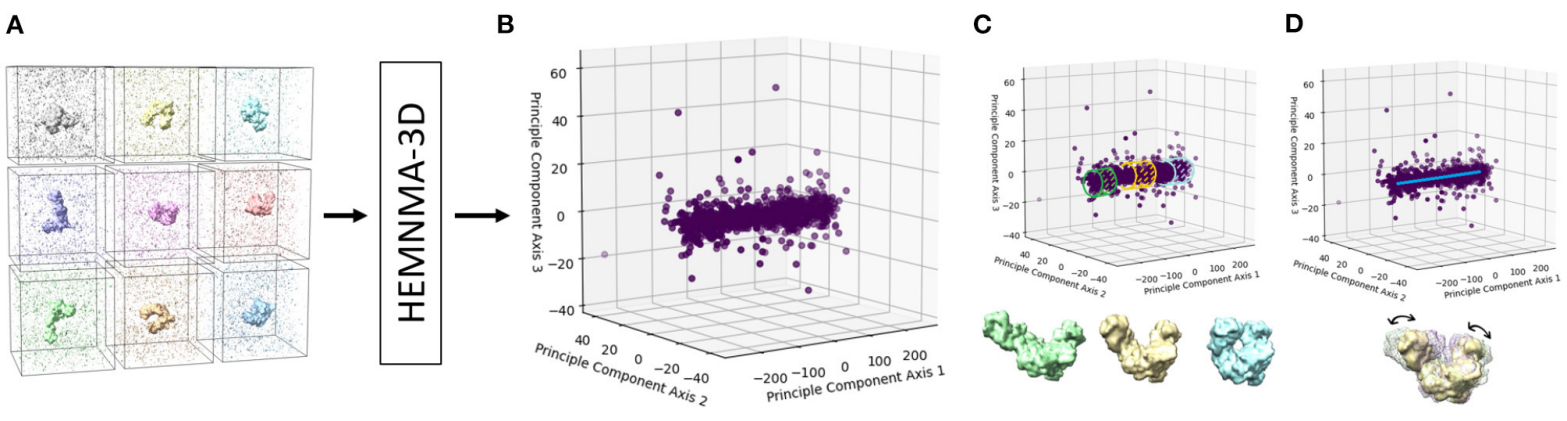
A graphical summary of the dataflow of HEMNMA-3D. **(A)** Input subtomograms containing the same biomolecule but at different orientations, positions and conformations (here represented with a low level of noise for illustration). **(B)** Input subtomograms projected onto a low-dimensional “space of conformations,” describing and visualizing the biomolecular conformational variability contained in the subtomograms. **(C)** Grouping of close points (subtomograms with similar biomolecular conformations) and averaging of subtomograms in these groups. **(D)** Animating biomolecular motion along trajectories identified in the densest regions.

The flowchart in Figure 2 describes the workflow of the proposed method, which was inspired by the workflow of HEMNMA (Jin et al., 2014; Harastani et al., 2020). A graphical summary of the method is presented in Figure 3. The workflow comprises the following steps: (1) Input: the input to the method are a reference structure and a set of subtomograms. In the case where the reference structure is a density map (a 3D volume, such as an EM map or a subtomogram average), a conversion to 3D Gaussian functions (pseudoatoms) takes place. (2) Normal mode analysis of the reference atomic structure or the reference pseudoatomic structure (obtained by converting the reference density map into 3D Gaussian functions in the previous step). (3) Combined iterative elastic and rigid-body 3D-to-3D alignment of the reference structure with each input subtomogram independently from other subtomograms, with missing wedge compensation. (4) Visualization of the computed conformations, after projecting the conformational parameters obtained for all subtomograms onto a low-dimensional space. In the remaining part of this section, we describe these steps in more detail. Please note that the first two steps of the workflow are exactly as those of HEMNMA and were thoroughly presented, tested and discussed in our previously published works on HEMNMA, its tools and applications (Nogales-Cadenas et al., 2013; Jin et al., 2014; Sorzano et al., 2014; Jonić and Sorzano, 2016; Harastani et al., 2020). However, for completeness of the present article, we here recall their basic principles. We close this section by a brief description of the software implemented for HEMNMA-3D.

### 2.1. Input Reference and Conversion of Reference Density Maps Into Pseudoatoms

A reference structure of the molecule targeted in the subtomograms can be used in the form of an atomic model (PDB formatted files) or a density map, such as an EM map (SPA reconstruction) or a subtomogram average (obtained using classical StA without taking into account conformational heterogeneity). Although our method can be used with both atomic and density-map reference structures, one should prefer the use of a reference density map from the data at hand, if it can be obtained. If a reference density map is used, it must be converted into a collection of Gaussian functions (pseudoatoms) with a carefully selected standard deviation (pseudoatom size, whose default value is 1 voxel Nogales-Cadenas et al., 2013; Jonić and Sorzano, 2016). The pseudoatom size should lead to a structure (called pseudoatomic structure) that, converted back to a density map, approximates the input density map with a small error (given a target approximation error, whose default value is 5% Nogales-Cadenas et al., 2013; Jonić and Sorzano, 2016). Optionally, a mask on the density map can be used prior to the conversion into pseudoatoms (e.g., a spherical binary mask of a given radius) to reduce background noise. Such masks may also be useful if applied on input cryo-ET subtomograms to maximize the chance of having a single molecular complex in each subtomogram (Preprocessing block in the workflow in Figure 2A).

### 2.2. Normal Mode Analysis

This step involves computing normal modes of a reference atomic or pseudoatomic structure, for the 3D-to-3D elastic alignment in the next step. The computation of normal modes is based on the elastic network model (Tirion, 1996; Tama et al., 2002) by representing the interaction between the (pseudo-)atoms as if they are locally connected by elastic springs (within a cutoff distance). Normal Mode Analysis requires the diagonalization of a 3*N* × 3*N* matrix of second derivatives of the potential energy (Hessian matrix), where *N* is the number of nodes in the elastic network model determined by the total number of atoms (or pseudoatoms) in the input reference. In the case of atomic structures, we use the rotation-translation block (RTB) method, which divides the structure into blocks (one or a few consecutive residues per block) whose rotations and translations are considered rather than all degrees of freedom for all atoms (Durand et al., 1994; Tama et al., 2000). Since the RTB method reduces the basis for Hessian diagonalization, it allows fast computing of normal modes. Since pseudoatomic structures usually contain fewer nodes (pseudoatoms) than atomic structures, normal modes can be obtained by a direct diagonalization of the 3*N* × 3*N* Hessian, which is referred to as the Cartesian method. As in the case of HEMNMA, we here use the RTB and Cartesian method implementations of Tama et al. (2002) and Suhre and Sanejouand (2004), respectively. Larger values of the interaction cutoff distance (the distance below which atoms or pseudoatoms do not interact) lead to more rigid motions. The atomic interaction cutoff distance may be set manually (by default 8 Å) and the pseudoatomic cutoff distance is recommended to be computed automatically based on the distribution of the pseudoatomic pairwise distances (e.g., as the value below which is a given percentage of all distances as in Nogales-Cadenas et al., 2013; Jin et al., 2014; Jonić and Sorzano, 2016; Harastani et al., 2020). The modes are computed along with their respective collectivity degrees, which count the number of atoms or pseudoatoms affected by the mode as in Brüschweiler (1995). To allow faster data analysis and avoid noise overfitting in the 3D-to-3D elastic alignment in the next step, we select a subset of normal modes (usually, less than 10) with lowest frequencies and highest collectivities, as previously described (Jin et al., 2014; Sorzano et al., 2014; Harastani et al., 2020). Low-frequency high-collectivity normal modes have been shown to be relevant to functional conformational changes (Tama and Sanejouand, 2001; Delarue and Dumas, 2004; Wang et al., 2004; Ma, 2005; Suhre et al., 2006; Tama and Brooks, 2006). The first six (lowest-frequency) normal modes are related to rigid-body transformations and are thus not used for the 3D-to-3D elastic alignment in the next step. The rigid-body 3D-to-3D alignment is done without using these rigid-body normal modes, as explained in the next paragraph. Additionally, a prior knowledge about the conformational transitions of the complex under study can be used to select the normal modes for the use in the next step. The HEMNMA-3D graphical interface helps the user decide which normal modes to select. The reader is referred to Ma (2005) and Tama and Brooks (2006) for reviews on the usefulness and limitations of NMA.

### 2.3. Combined Iterative Elastic and Rigid-Body 3D-to-3D Alignment

This step, represented in Figure 2B, is the backbone of the proposed method. It has been inspired by the combined iterative elastic and rigid-body 3D-to-3D alignment step of StructMap method (Sorzano et al., 2016), which was proposed for pairwise similarity analysis of SPA high-resolution EM maps (no missing wedge). In HEMNMA-3D proposed here, this step comprises simultaneous NMA-based elastic alignment (search for amplitudes of a linear combination of normal modes) and rigid-body alignment (search for orientation and position, meaning three Euler angles and x, y, and z shifts) of the reference structure with each given subtomogram. It refines the amplitudes of displacement along each used normal mode (elastic parameters) as well as the angles and shifts (rigid-body parameters) of the reference structure until the best match is obtained between this reference structure and the given subtomogram. The latter is achieved by maximizing the similarity between the subtomogram and the density volume from the elastically deformed, oriented and shifted reference, and includes missing wedge compensation. The missing wedge compensation is done by calculating the cross-correlation between the reference and subtomogram density maps only in the region of the Fourier space where the data can be trusted, i.e., by constraining the cross-correlation evaluation to the Fourier space region that excludes the missing wedge region (the region outside of the one specified by the tilt angle range, e.g., −60 to +60°). To maximize this constrained cross-correlation (CCC), we use a variant of Powell's UOBYQA method, which subjects the objective function to a trust-region radius (Berghen and Bersini, 2005). To control the elastic deformation with highly noisy data, the radius of the trust region is adjusted iteratively. The scaling factor of the initial trust-region radius is a parameter that controls the normal-mode amplitude search range and can be modified by the user. It should have a positive value and its default value of 1 produces good results in general. It may be increased (typically to a value between 1 and 2) or decreased (e.g., between 0.5 and 0.9), if expecting larger or smaller conformational changes, respectively. For each subtomogram, the normal mode amplitudes are initiated with zeros, meaning that the non-deformed reference is used in the first iteration. As the iterations evolve, the reference model is displaced with the new guesses of the normal mode displacement amplitudes, converted into a volume and rigid-body aligned with the subtomogram using the method of fast rotational matching. Fast rotational matching has been largely used for rigid-body fitting of atomic models to high-SNR consensus EM maps (Kovacs and Wriggers, 2002; Kovacs et al., 2003). It has been extended to alignment of noisy subtomograms in Chen et al. (2013) and this implementation is used in our work. At the end of each iteration, the CCC is found and fed to the numerical optimizer (Berghen and Bersini, 2005). The iterations repeat until the final value of the trust-region radius or the maximum number of iterations is reached.

### 2.4. Visualizing and Utilizing the Space of Conformations

The number of elastic alignment parameters (normal mode amplitudes) is determined by the number of selected normal modes for the 3D-to-3D elastic alignment. The ensemble of normal mode amplitudes (for all subtomograms) can be projected onto a lower-dimensional space, so-called conformational space, using a dimensionality reduction technique. Here, we use linear Principal Component Analysis (PCA) as it is the most widely known and intuitively clear dimensionality reduction method, but other dimension reduction methods could also be used (linear or nonlinear). The dimensionality reduction is usually performed to two or three dimensions, which allows a global data display and easier modeling of conformational changes. Each point in the conformational space represents a subtomogram and close points correspond to similar conformations in the subtomograms. The points that differ significantly from the remaining observations (too isolated, outlier points) may be excluded from the further analysis, by excluding the points below a certain p-value based on the Mahalanobis distance (the distance between each point and the whole distribution) (Mahalanobis, 1936). The excluded points can be explained by the fact that some orientations of the molecule combined with the missing wedge artifacts and the high noise make some volumes more difficult to align with the elastically deformed reference. After excluding such outlier points, the space of conformations can be analyzed to reveal molecular dynamics. This can be done by averaging subtomograms of similar conformations in the densest regions of the conformational space or by exploring the densest regions by fitting curves (approximation by line segments) through the data and displacing the reference structure along these curves (referred to as trajectories) to animate the motion along them. The 1-CCC color bar of the conformational space shows coloring the points according to the value of the CCC between the subtomograms and the density maps from the elastically deformed reference model. Subsequently, the colors provide the level of confidence in the obtained conformations. Those subtomograms in which we have less confidence (subtomograms with lower CCC values) than in the “consensus” observations (subtomograms with higher CCC values) can be eliminated from the group averages.

#### 2.4.1. Averaging Subtomograms of Similar Conformations

Close points in the conformational space can be grouped, which results in grouping subtomograms of similar conformations and averaging them. Before computing group averages, the rigid-body alignment parameters found during the combined iterative elastic and rigid-body alignment are applied on the subtomograms. Optionally, before computing group averages, the missing-wedge Fourier space region of individual subtomograms may be filled in with the corresponding region of the global average computed from all subtomograms. A similar procedure of missing wedge filling of individual subtomograms is used in EMAN2 software package (Galaz-Montoya et al., 2015). The subtomogram averages obtained from the selected groups of subtomograms can be overlapped and compared to understand the conformational changes of the complex in the given set of subtomograms.

#### 2.4.2. Animating Motions (Trajectories)

Distinct trajectories can be determined through the data in the conformational space, and animated to see the motion of the biomolecule while it is displaced along the trajectory. To animate a trajectory, several points (e.g., 10) along the trajectory should be mapped back to the original displacement space (e.g., using inverse PCA), resulting in elastic alignment parameters that can be used to deform the reference atomic or pseudoatomic structure. Concatenating and displaying the resulting structures can show a movie-like animation of the reference biomolecule traveling across the specified trajectory.

### 2.5. Software Implementation and Technical Details

The software of HEMNMA-3D method proposed here is freely available (open-source). It is a part of ContinuousFlex plugin for the open-source software Scipion3 (De la Rosa-Trev́ın et al., 2016). ContinuousFlex was introduced in Harastani et al. (2020) and also contains HEMNMA software. The software provides a graphical user interface (GUI) and is empowered with a C++ backend with a message passing interface (MPI) parallelization scheme to efficiently analyze large datasets (simultaneous analysis of *N* subtomograms using *N* computing threads). We tested the software on our local workstations and on supercomputer centers. On our local workstations (2.2 GHz Intel Xeon Silver 4214 CPU processors), the current implementation takes around 10 and 30 min to analyze a subtomogram of size 64^3^ voxels with three and six normal modes, respectively. These times are reported for the two types of complexes used in this article, together with the number of normal modes used in these two cases. They are the average times required for all iterations of analyzing one subtomogram using a single computing thread while the different subtomograms are analyzed in parallel using different computing threads (if the time is measured for the entire dataset, it will vary with the size of the dataset). It should be noted that the software allows the use of any number of MPI threads and any number of normal modes. However, the more modes are used, the slower the processing. Finally, it should be noted that there is no constraint regarding the size of the dataset (the number of subtomograms) or the size of the individual subtomograms (the number of voxels) that can be analyzed with our software.

## 3. Results and Discussion

In this section, we present and discuss the results of HEMNMA-3D with synthetic and experimental subtomograms.

### 3.1. Synthesizing Datasets for Testing the Method Performance

**Figure 4:**
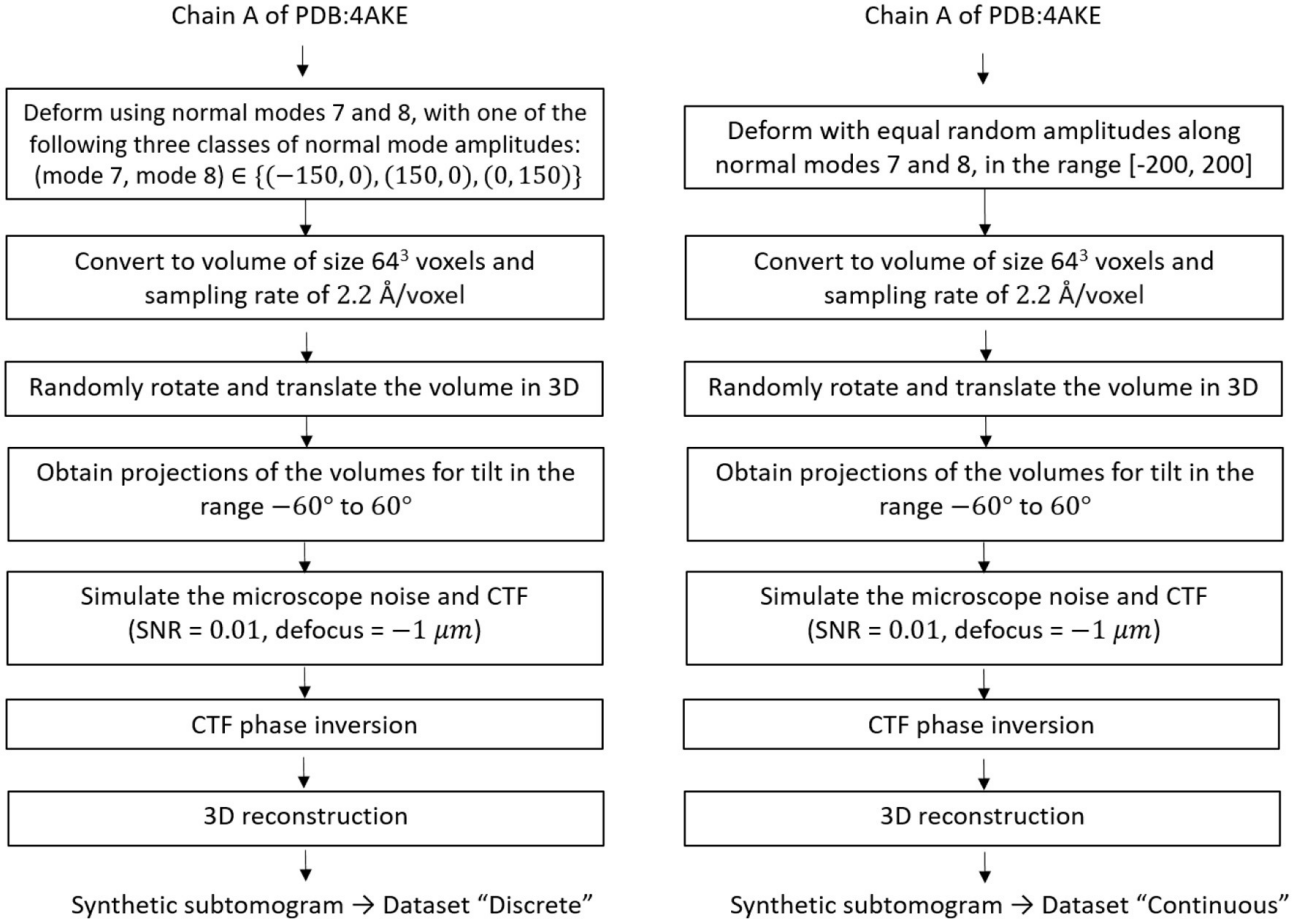
Flowcharts of synthesis of the datasets used for testing and validating HEMNMA-3D, namely “Discrete” dataset (left) and “Continuous” dataset (right).

For testing HEMNMA-3D in general, and the combined elastic and rigid-body 3D-to-3D alignment module in particular (which is the core module of the proposed method), we synthesized two datasets of conformationally heterogeneous subtomograms that mimic discrete and continuous conformational variability, called “Discrete” and “Continuous” datasets, respectively. The flowchart for the data generation procedure is shown in Figure 4 and is detailed in the following.

The “Discrete” dataset comprises 900 synthetic subtomograms representing three different (synthetic) conformations of the atomic PDB:4AKE structure (Müller et al., 1996) of adenylate kinase chain A (1656 atoms), i.e., 300 subtomograms per conformation. We generated this dataset using the atomic PDB:4AKE structure and its first two non-rigid-body normal modes, i.e., modes 7 and 8. Precisely, the three conformations are represented by the following amplitudes of modes 7 and 8: (mode 7, mode 8) ∈ {(−150, 0), (+150, 0), (0, +150)}.

The “Continuous” dataset comprises 1,000 synthetic subtomograms representing a continuum of conformations of the same PDB:4AKE structure. We generated this dataset using this atomic structure and its modes 7 and 8 using a linear relationship between the amplitudes of the two modes. More precisely, the synthesized amplitudes of modes 7 and 8 were identical and randomly distributed in the range [−200, +200] (uniform distribution).

Normal-mode amplitudes do not have a physical unit. Nonetheless, the Root Mean Square Deviation (RMSD) (Kufareva and Abagyan, 2011) between the reference atomic coordinates and these coordinates displaced using normal-mode amplitudes transforms the normal mode amplitudes in physical units. To provide a basis for further evaluation of the method performance, we found a RMSD of 6.95 Å corresponding to the displacement using the amplitude of 200 for each of the two combined modes 7 and 8 (this represents one half of the full range of the synthesized motion).

**Figure 5:**
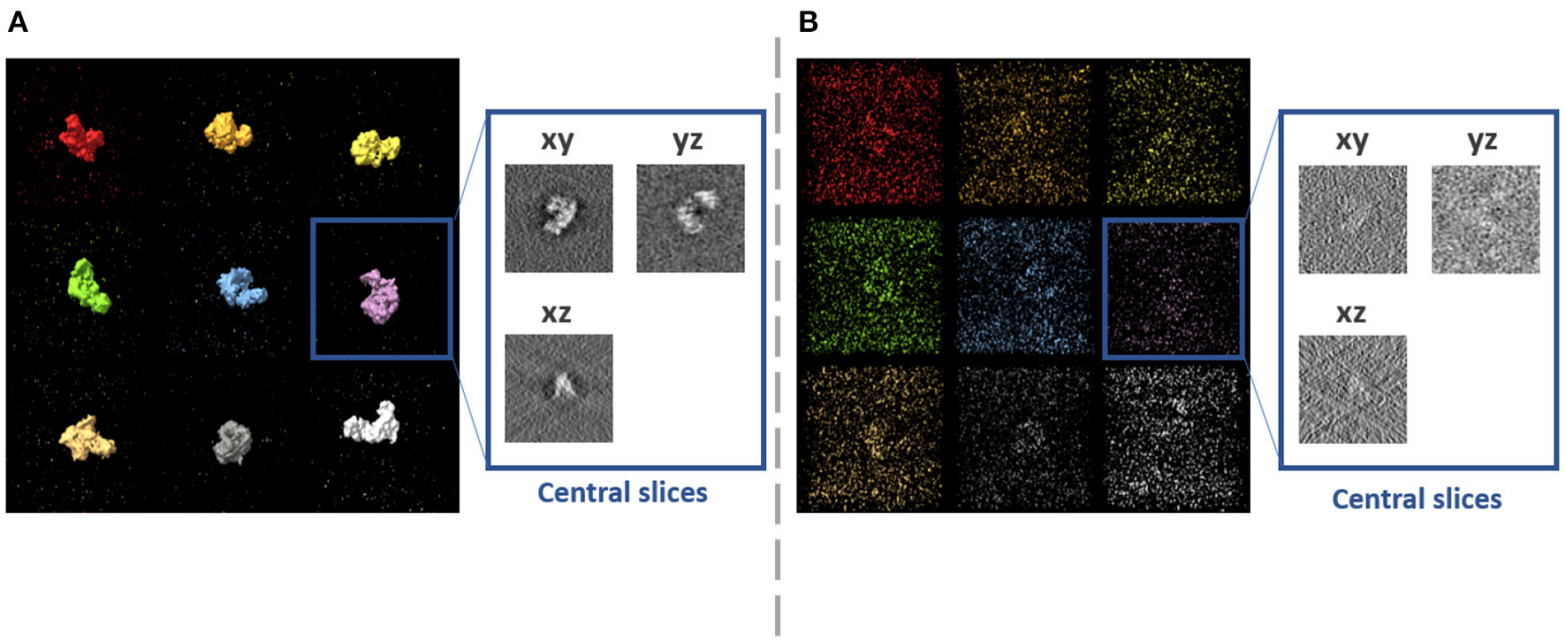
Examples of synthetic subtomograms containing the same molecule but at different orientations, positions and conformations, for two different noise levels. **(A)** Low level of noise (SNR = 0.5). **(B)** High level of noise (SNR = 0.01).

To generate a subtomogram, first, we deform the atomic structure using appropriate amplitudes for the selected normal modes depending on the dataset in hand, i.e., we use (mode 7, mode 8) = (+150, 0) or (−150, 0) or (0, +150) to create a subtomogram in the “Discrete” dataset, while we assign a random value in the range [−200, 200] for both mode 7 and mode 8 to generate a subtomogram in the “Continuous” dataset. Then, we convert the deformed structure to a volume of size 64^3^ voxels and the voxel size of 2.2 Å^3^ (Peng et al., 1996). Afterwards, we rotate and shift this volume in 3D space using random Euler angles (each of the three Euler angles was randomized in the range [0, 360°]) and random shifts (the shift along each of the x, y, and z axes was randomized in the range [−5, +5] voxels), and we project the rotated and shifted volume using tilt values −60 to +60° to obtain a tilt series. We simulate microscope conditions by adding heavy noise (signal to noise ratio SNR = 0.01) and modulating the images with a contrast transfer function (CTF) of defocus −1 μm, so that one part of the noise is affected by the CTF and the other is not (Sorzano et al., 2007; Chen et al., 2013). Finally, we reconstruct a volume (our synthetic subtomogram) from the tilt series using a Fourier reconstruction method (Sorzano et al., 2013). A few examples of the synthesized subtomograms (SNR = 0.01) and their less noisy version (SNR = 0.5, for illustration) is presented in Figure 5.

### 3.2. Synthetic Discrete-Type Conformational Variability

**Figure 6:**
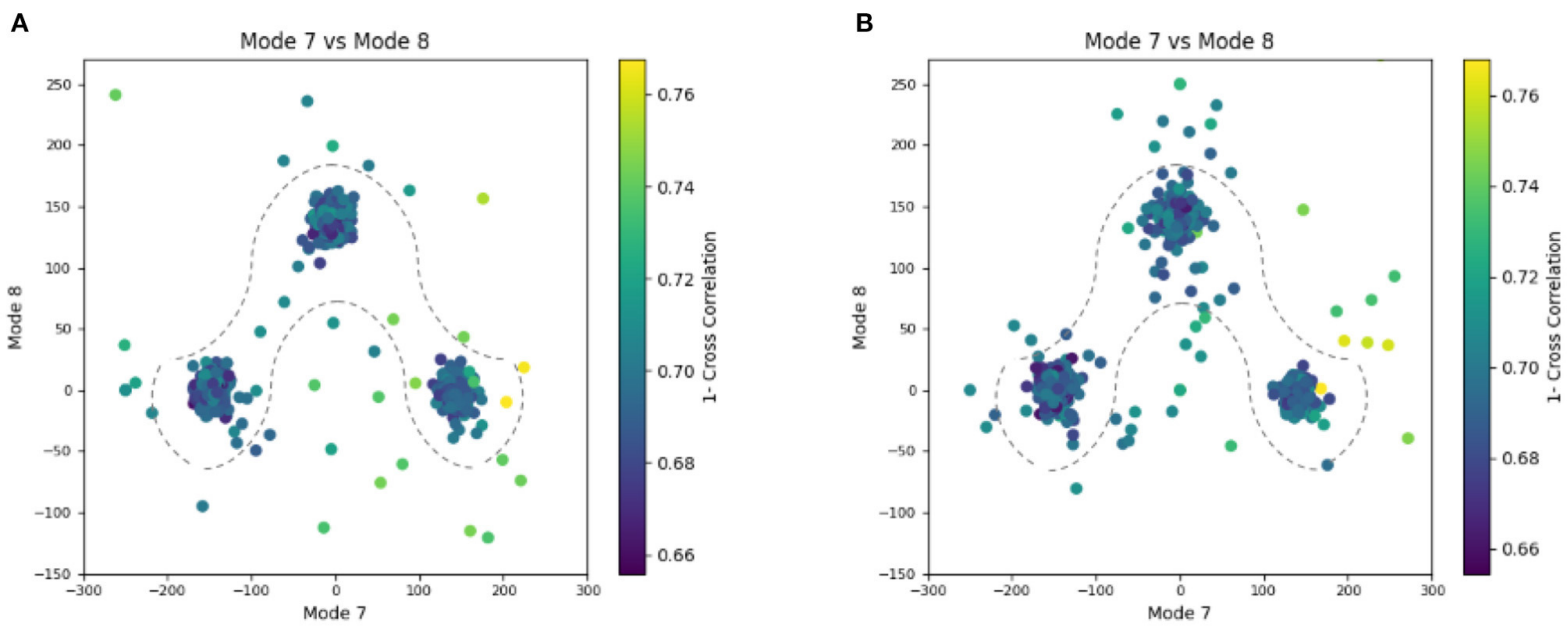
Plots showing the output of the 3D-to-3D elastic and rigid-body alignment module of HEMNMA-3D with “Discrete” dataset (synthetic subtomograms are simulating discrete conformational heterogeneity). **(A)** Use of the atomic structure (chain A of PDB:4AKE) and its normal modes to estimate the conformational parameters (normal-mode amplitudes) and rigid-body parameters (orientation and shift) of the molecules in the input synthetic subtomograms. **(B)** Use of a pseudoatomic structure (from a simulated density map) and its normal modes to estimate the conformational and rigid-body parameters of the molecules in the input synthetic subtomograms. The goal was the retrieval of the ground-truth relationship between the amplitudes along normal modes 7 and 8; ideally, all data should lay in one of the following three clusters of normal-mode amplitudes: (mode 7, mode 8) ∈ {(-150, 0), (150, 0), (0, 150)}; each point in the plot represents a subtomogram and close points represent similar conformations. Note that the dashed curves enclose the data points where *p*-value > 0.01 in Table 1. See the text for more details on this experiment.

**Figure 7:**
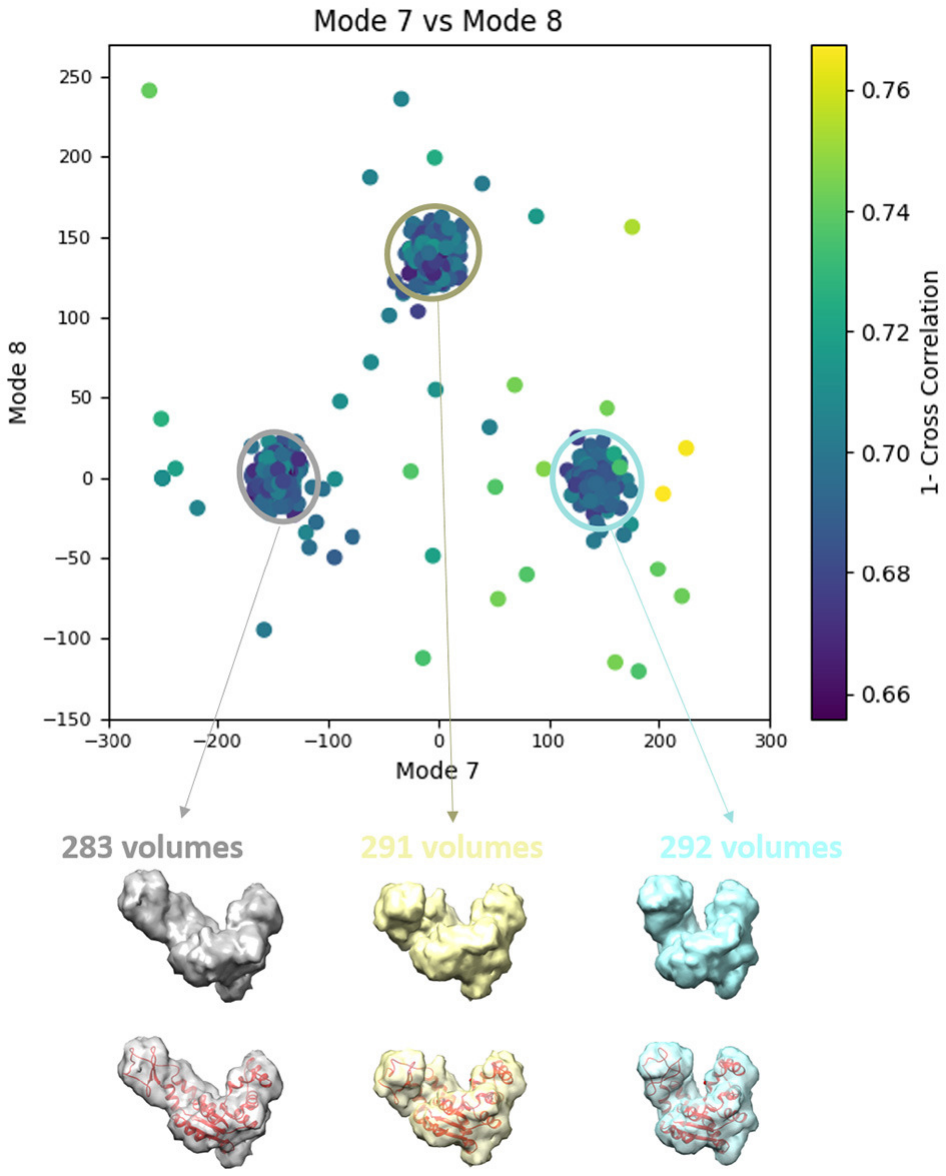
Averages of the three groups (enclosed by ellipses) of subtomograms identified from the output of the 3D-to-3D elastic and rigid-body alignment module of HEMNMA-3D with “Discrete” dataset (shown in Figure 6A), using the atomic structure (chain A of PDB:4AKE) and its normal modes to estimate the conformational parameters (normal-mode amplitudes) and rigid-body parameters (orientation and shift) of the molecules in the input synthetic subtomograms. Subtomograms are represented by points and close points represent similar conformations. The numbers of volumes written above the shown subtomogram averages are the numbers of synthetic subtomograms used for computing these subtomogram averages (the numbers of points enclosed by the corresponding ellipses). On the bottom, the subtomogram averages are shown at 50% transparency along with the corresponding ground-truth deformed atomic structure (in red).

In this experiment, our goal is to retrieve the ground-truth amplitudes of normal modes 7 and 8 by the combined elastic and rigid-body alignment (the core module of HEMNMA-3D) of a reference model with the subtomograms in the “Discrete” dataset. In other words, the goal is to find a solution for the challenging inverse problem of finding the conformation of the structure in each subtomogram. Since the proposed method can use two choices for the reference model, namely, an atomic structure and a density map (e.g., an EM map or a subtomogram average), we performed two types of tests. In the first test type, the atomic structure used to generate the synthetic subtomograms (chain A of the PDB:4AKE) was used as a reference for retrieving normal mode amplitudes of the synthetic subtomograms. In the second test type, we converted (Peng et al., 1996) the atomic structure into a density map (volume) of size 128^3^ voxels and voxel size of 1 Å^3^, and we used this density map as a reference for retrieving normal mode amplitudes of the synthetic subtomograms. In the case of the reference density map, normal modes were computed from the corresponding structure obtained by converting the density map into pseudoatoms (1675 pseudoatoms for the given pseudoatom radius of 1.25 voxels and the target approximation error of 5%). In both cases (reference atomic structure and reference pseudoatomic structure, with their corresponding normal modes), we used three modes (modes 7, 8, and 9) instead of only two modes (modes 7 and 8 that were used to generate synthetic subtomograms), to make the 3D-to-3D elastic and rigid-body alignment task even more challenging. Figure 6 presents the estimated amplitudes of normal modes 7 and 8 (the estimated amplitude of normal mode 9 is close to 0 and is therefore not shown graphically). Table 1 presents the mean absolute error and the standard deviation between the estimated and ground-truth normal-mode amplitudes along with the angular and shift distances. In both test cases, the three distinct synthetic groups of subtomograms are correctly separated, taking into account the extreme noise level. The results show a less accurate alignment in the second case, which is expected since, in that case, the atomic structure was used to generate the dataset and the pseudoatomic structure was used as the reference model for the method to estimate the normal-mode amplitudes from this generated dataset. This is in contrast to the first test case where the same atomic structure was used to create the dataset and as the reference for the method to estimate the normal-mode amplitudes from this dataset. We found a RMSD of 0.55 and 0.60 Å corresponding to a combined displacement along modes 7, 8, and 9 with the mean absolute errors in Table 1 for the tests with atomic and pseudoatomic structures, respectively. Similarly, we found a RMSD of 0.94 and 1.06 Å corresponding a combined displacement along modes 7, 8, and 9 with the sum of the mean and standard deviation of the absolute errors in Table 1 for the tests with atomic and pseudoatomic structures, respectively. Hence, the error range is significantly inferior to the half range of the synthesized motion (6.95 Å) and the pixel size used to create the data (2.2 Å). Figure 7 shows grouping and averaging the subtomograms in the first test type (atomic reference). We compared the obtained subtomogram averages with the corresponding ground-truth volumes (density maps from ground-truth deformed models, without noise and missing wedge, used for synthesizing noisy and CTF-affected tilt-series from which subtomograms were obtained by 3D reconstruction). The visual comparison shows no significant difference between them. The resolutions calculated using Fourier shell correlation—FSC (threshold value of 0.143), after applying onto the subtomogram average a large spherical mask (radius of 28 voxels) with smooth edges (Gaussian smoothing with Gaussian standard deviation of five voxels), are 5.30, 5.35, and 5.74 Å for the three subtomogram averages shown from left to right in Figure 7, respectively. Without masking subtomogram averages, these resolutions are 6.31, 6.10, and 6.43 Å, respectively. As a basis for comparison, we provide the resolutions of three individual subtomograms arbitrarily chosen from the three corresponding subtomogram averaging groups. The resolutions of individual subtomograms with masking (the mask already described) are 10.58, 10.57, and 12.75 Å. The resolutions of the same subtomograms without masking are 12.67, 13.55, and 14.72 Å. These results show that a twice better resolution is obtained after averaging only about 300 individual subtomograms per group (Figure 7).

**Table 1:** Mean absolute error and standard deviation between the estimated and ground-truth normal-mode amplitudes along with the angular and shift distances obtained with HEMNMA-3D and “Discrete” synthetic dataset, using an atomic structure (Atomic) and simulated EM map (Volume) as input references.

**Experiment**		**Mode 7**		**Mode 8**		**Mode 9**		**Angular (deg)**		**Shifting (vox)**		***p*-Value**	**Samples**
**Ref**	**Dataset**	**Mean**	**Std**	**Mean**	**Std**	**Mean**	**Std**	**Mean**	**Std**	**Mean**	**Std**		
Atomic	“Discrete”	16.51	11.87	10.91	7.64	10.70	6.66	1.33	0.77	0.19	0.09	*p* > 0.01	871/900
Volume	“Discrete”	17.70	13.26	11.90	10.13	12.29	8.03	1.33	0.81	0.21	0.10	*p* > 0.01	870/900

*The data points below the p-value of 0.01 were excluded from the error evaluation based on the Mahalanobis distance measure (few data points differing significantly from the remaining observations, which would not be selected in real-case experiments as being too isolated and far from other points). The number of points used for the error computation is shown in the last column of the table (column Samples) and the region with the kept points (p-value > 0.01) is shown in Figure 6*.

### 3.3. Synthetic Continuous-Type Conformational Variability

**Figure 8:**
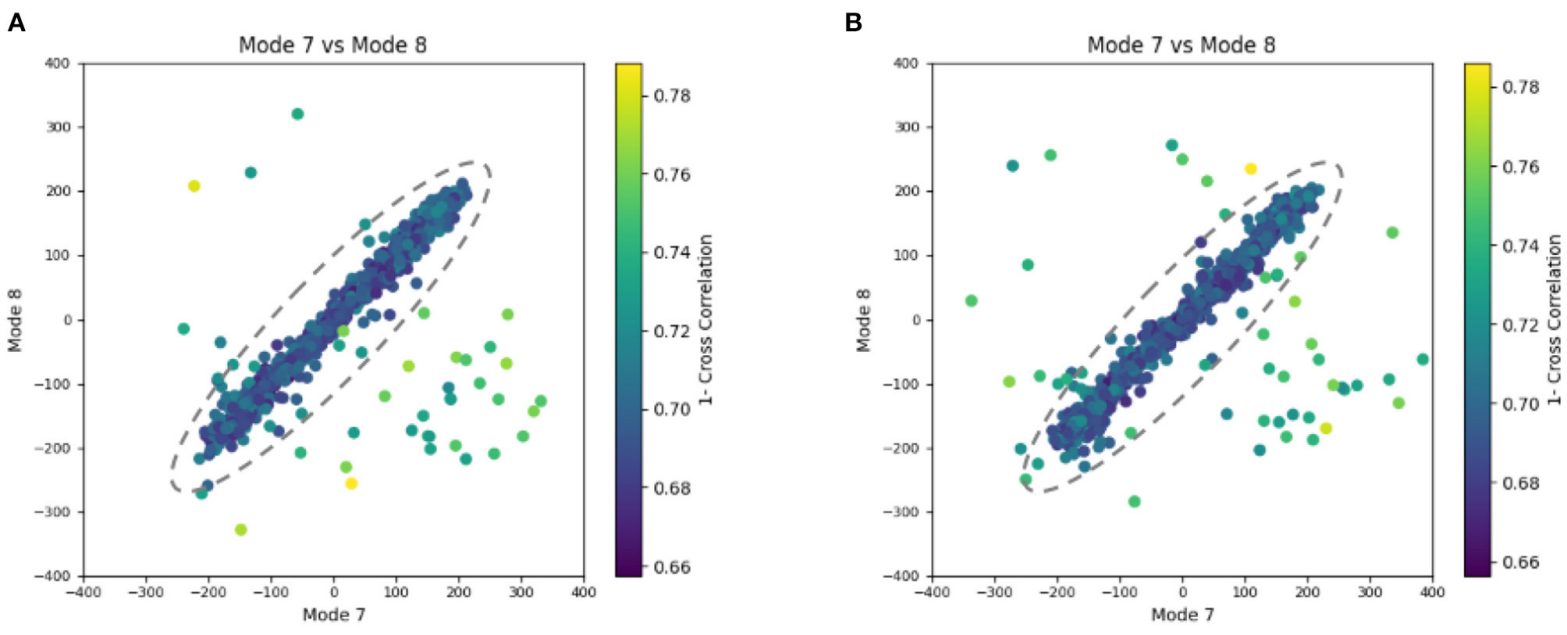
Plots showing the output of the 3D-to-3D elastic and rigid-body alignment module of HEMNMA-3D with “Continuous” dataset (synthetic subtomograms are simulating continuous conformational heterogeneity). **(A)** Use of the atomic structure (chain A of PDB:4AKE) and its normal modes to estimate the conformational parameters (normal-mode amplitudes) and rigid-body parameters (orientation and shift) of the molecules in the input synthetic subtomograms. **(B)** Use of a pseudoatomic structure (from a simulated density map) and its normal modes to estimate the conformational and rigid-body parameters of the molecules in the input synthetic subtomograms. The goal was the retrieval of the ground-truth relationship between the amplitudes along normal modes 7 and 8 (ideally linear relationship, with equal amplitudes of normal modes 7 and 8); each point in the plot represents a subtomogram and close points represent similar conformations. Note that the dashed ellipses enclose the data points where *p*-value > 0.001 in Table 2. See the text for more details on this experiment.

**Figure 9:**
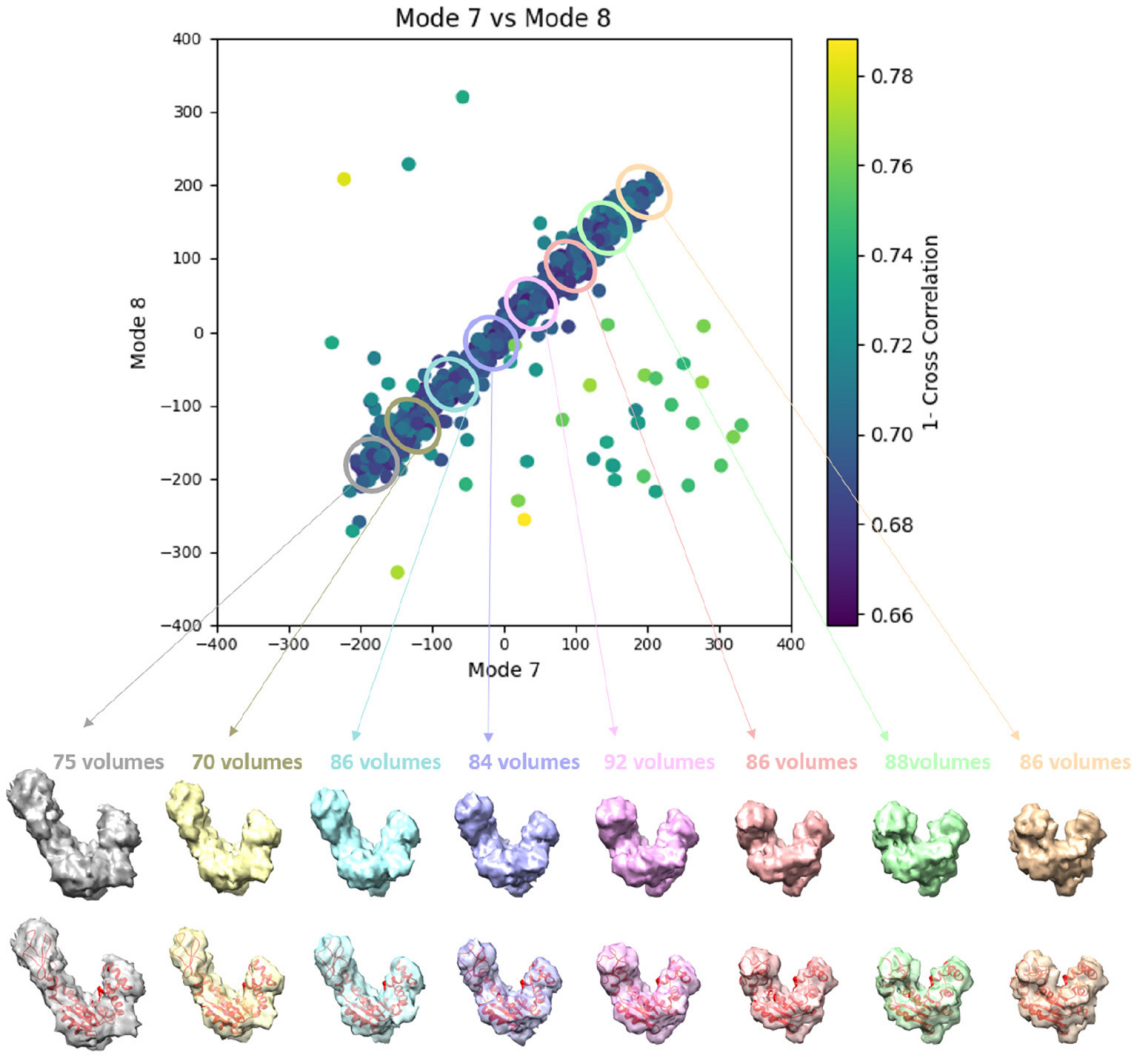
Averages of eight groups (enclosed by ellipses) of subtomograms identified from the output of the 3D-to-3D elastic and rigid-body alignment module of HEMNMA-3D with “Continuous” dataset (shown in Figure 8A), using the atomic structure (chain A of PDB:4AKE) and its normal modes to estimate the conformational parameters (normal-mode amplitudes) and rigid-body parameters (orientation and shift) of the molecules in the input synthetic subtomograms. Subtomograms are represented by points and close points represent similar conformations. The numbers of volumes written above the shown subtomogram averages are the numbers of synthetic subtomograms used for computing these subtomogram averages (the numbers of points enclosed by the corresponding ellipses). On the bottom, the subtomogram averages are shown at 50% transparency along with the corresponding theoretical centroid deformed atomic structure (in red).

Similarly to the previous experiment, our goal in this experiment is to find a solution for the inverse problem of finding the conformation of the structure in each subtomogram using the combined elastic and rigid-body alignment of a reference model with the subtomograms in the “Continuous” dataset. We used the same two reference models as in the previous experiment to estimate the normal-mode amplitudes: an atomic structure (chain A of PDB:4AKE) and a density map from this atomic structure. Also, as in the previous experiment, we used three modes for both tests (atomic or pseudoatomic modes 7, 8, and 9). Figure 8 presents the estimated amplitudes of modes 7 and 8 (the estimated amplitude of mode 9 is close to 0 and is not shown in the plots). Table 2 shows the mean absolute error and the standard deviation between the estimated and ground-truth normal-mode amplitudes along with the angular and shift distances. In both test cases, a linear relationship between the estimated amplitudes of normal modes 7 and 8 is clearly distinguishable, which is close to the identity relationship between the ground-truth amplitudes taking into account strong noise present in the data. As in the previous experiment, the results show a slightly less accurate alignment in the second test type (pseudoatomic reference) for the same aforementioned reason. We found a RMSD of 0.66 and 0.75 Å corresponding to a combined displacement along modes 7, 8, and 9 with the mean absolute errors in Table 2 for the tests with atomic and pseudoatomic structures, respectively. Similarly, we found a RMSD of 1.09 and 1.21 Å corresponding a combined displacement along modes 7, 8, and 9 with the sum of the mean and standard deviation of the absolute errors in Table 2 for the tests with atomic and pseudoatomic structures, respectively. Hence, the error range is significantly inferior to the half range of the synthesized motion (6.95 Å) and the pixel size used to create the data (2.2 Å). Figure 9 shows grouping and averaging of subtomograms in this experiment, with eight subtomogram averages calculated along the distribution of the points for the first test type (atomic reference). The subtomogram averages show different conformations of adenylate kinase chain A. Note that the noise contained in the individual subtomograms (SNR = 0.01, Figure 5B) was reduced through subtomogram averaging (Figure 9). Additional experiments, for other noise levels in input subtomograms, can be found in Supplementary Figure 1 and Supplementary Table 1.

**Table 2:** Mean absolute error and standard deviation between the estimated and ground-truth normal-mode amplitudes along with the angular and shift distances obtained with HEMNMA-3D and “Continuous” synthetic dataset, using an atomic structure (Atomic) and simulated EM map (Volume) as input references.

**Experiment**		**Mode 7**		**Mode 8**		**Mode 9**		**Angular (deg)**		**Shifting (vox)**		***p*-Value**	**Samples**
**Ref**	**Dataset**	**Mean**	**Std**	**Mean**	**Std**	**Mean**	**Std**	**Mean**	**Std**	**Mean**	**Std**		
Atomic	“Continuous”	20.12	11.30	12.78	11.24	12.74	7.71	1.31	0.79	0.19	0.10	*P* > 0.001	960/1,000
Volume	“Continuous”	21.94	12.59	14.03	9.92	15.68	10.04	1.34	0.80	0.21	0.10	*P* > 0.001	957/1,000

*The data points below the p-value of 0.001 were excluded from the error evaluation based on the Mahalanobis distance measure (few data points differing significantly from the remaining observations, which would not be selected in real-case experiments as being too isolated and far from other points). The number of points used for the error computation is shown in the last column of the table (column Samples) and the region where p-value > 0.001 is shown in Figure 8*.

### 3.4. Experimental Cryo-ET Data: Nucleosomes *in situ*

**Figure 10:**
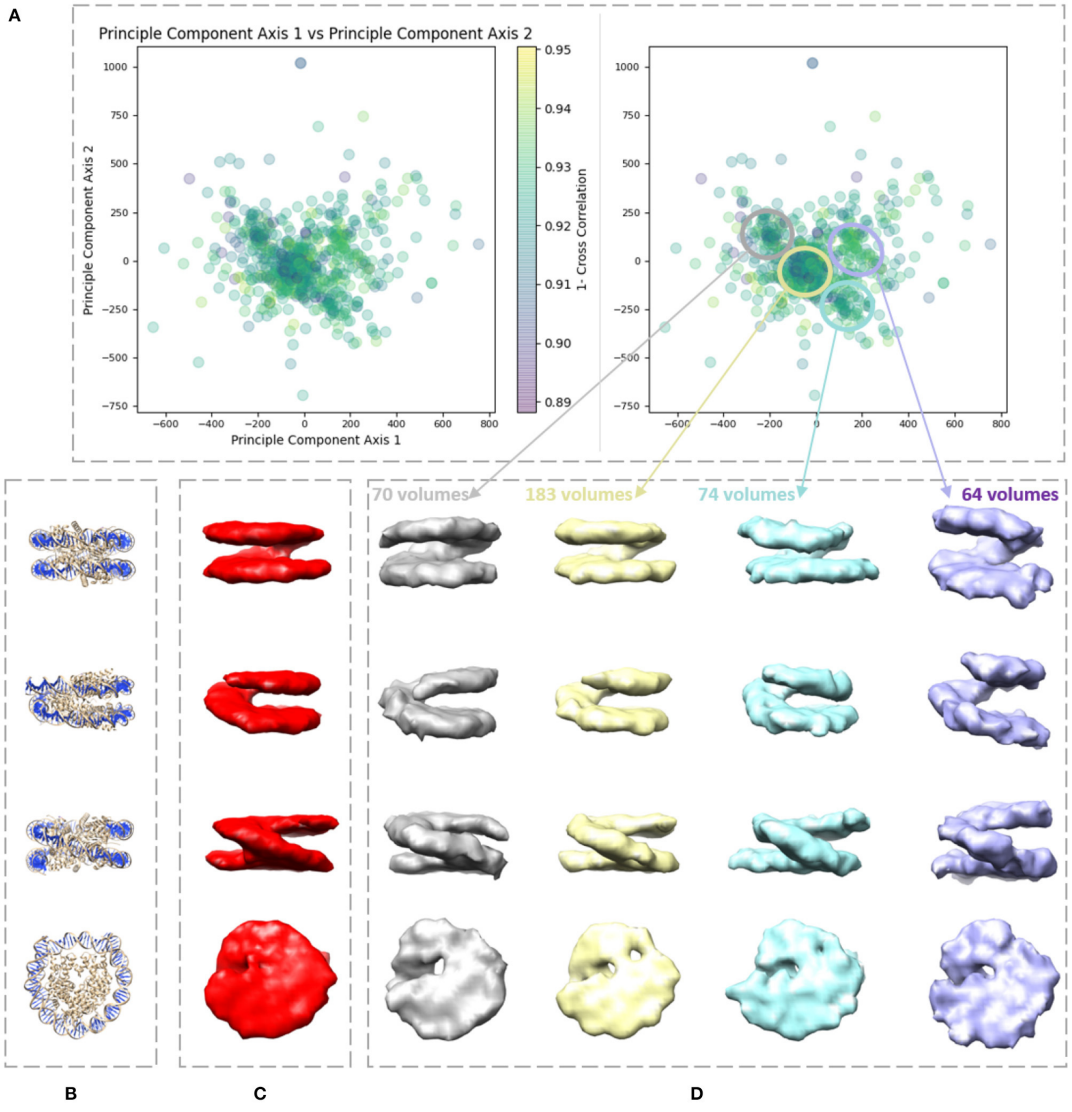
Illustration of HEMNMA-3D use with *in situ* cryo-ET nucleosome dataset. **(A)** Space of conformations resulting from projecting the estimated amplitudes of six normal modes onto a two-dimensional space using PCA. **(B)** Nucleosome atomic structure PDB:3w98, for comparison purposes. **(C)** Nucleosome subtomogram average (around 2 nm resolution) used as the input reference density map for HEMNMA-3D, obtained by classical subtomogram averaging, without taking into account conformational heterogeneity [for more information on how this global initial subtomogram average was obtained, see section 1 of the Supplementary Material (Nucleosome data preparation and acquisition)]. **(D)** Four subtomogram averages from four densest regions in the space of conformations (regions encircled with ellipses) showing different nucleosome conformations, mainly, different gap distances between the nucleosome gyres. The numbers of volumes written above the subtomogram averages shown in **(D)** are the numbers of *in situ* cryo-ET subtomograms used for computing these subtomogram averages (the numbers of points enclosed by the corresponding ellipses).

**Figure 11:**
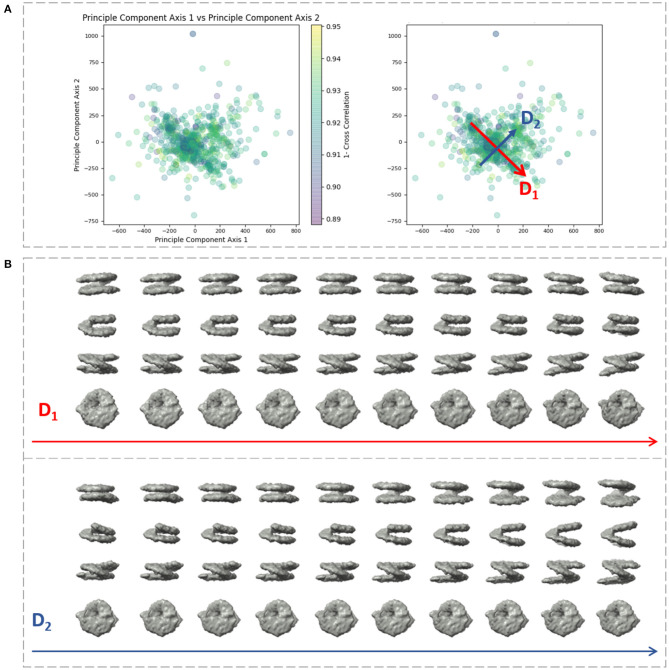
Displacement of the reference density map along two directions D1 and D2 in the space of conformations obtained (Figure 10) with HEMNMA-3D with *in situ* cryo-ET nucleosome dataset. **(A)** Space of conformations (left) as shown in Figure 10 and two directions D1 and D2 used to displace the reference density map (Figure 10C) in this space (right). **(B)** Displacement of the reference density map along the D1 and D2 directions (10 frames of the corresponding trajectory are shown row-wise).

We applied our method on a dataset comprising 650 *in situ* subtomograms of nucleosomes collected from a cell of a Drosophila embryonic brain, whose conformational variability was detected but not fully explored in a previous work (Eltsov et al., 2018). The subtomograms had the size of 64^3^ voxels and the voxel size of 4.4 Å^3^. A density map obtained with classical subtomogram averaging (without taking into account conformational heterogeneity) was used as the reference density map for HEMNMA-3D (Figure 10C). The resolution of this reference density map is around 2 nm (as determined by Fourier Shell Correlation between the reference density map and the density map from the atomic nucleosome structure PDB:3w98 Iwasaki et al., 2013 shown in Figure 10B). For more information on how this reference density map (global initial subtomogram average) was obtained, please see section 1 of the Supplementary Material (Nucleosome data preparation and acquisition). This reference density map was converted into pseudoatoms (1368 pseudoatoms for the pseudoatom radius of 0.5 voxels and the target approximation error of 5%) and normal mode analysis of the obtained reference pseudoatomic structure was performed. The combined elastic and rigid-body alignment was performed using the pseudoatomic structure and a set of its six low-frequency high-collectivity normal modes. We selected modes 7–11 and mode 16, as described above and in our previous works (Jin et al., 2014; Sorzano et al., 2014; Harastani et al., 2020). Modes 7–11 were selected as being the five lowest-frequency non-rigid-body modes with collectivities above 0.5. They include the mode related to gaping motion (mode 7) and the mode related to breathing motion (mode 9), which have been described in previous nucleosome studies (Zlatanova et al., 2009; Eltsov et al., 2018). Mode 16 was selected as being related to a motion that could be potentially interesting but it is more complex (a slightly higher frequency motion), potentially including gaping- and breathing-like motions. The normal-mode amplitudes estimated through the alignment (six normal mode amplitudes per subtomogram) were then projected onto a 2D space of conformations using PCA. The space of conformations is presented in Figure 10. Recall that each of the points represents a subtomogram, and close points represent similar conformations. By inspecting this conformational space, we identified four densest regions with 70, 183, 74, and 64 points from left to right in Figure 10D. Following this analysis, we grouped the subtomograms in each of these four regions and averaged them. Before averaging, we filled in the missing-wedge Fourier space region of the individual subtomograms with the corresponding region of the global average computed from all subtomograms (please note that this global average was computed after aligning subtomograms using the rigid-body alignment parameters found along with the 3D-to-3D elastic alignment by HEMNMA-3D, which is a similar density map to the initial global average map shown in Figure 10C as both density maps result from averaging conformational heterogeneous subtomograms). The displacement of the reference pseudoatomic structure (converted into a density map) along two directions D1 and D2 in the space of conformations is shown in Figure 11 and in Supplementary Videos 1, 2. The significant difference between the four group averages (Figure 10D) and the reference density map (Figure 10C) as well as the motion observed along the two directions D1 and D2 (Figure 11, Supplementary Videos 1, 2) can be described, mainly in terms of opening the nucleosome by increasing the distance between the two gyres of the DNA superhelix. This result consents the previous findings, observed but not fully explored in a previous study (manual analysis) of the nucleosome conformational variability (Eltsov et al., 2018). The group averages are also compared with the atomic nucleosome structure PDB:3w98 in Supplementary Figure 2.

## 4. Discussion and Conclusions

This article presents HEMNMA-3D, the first cryo-ET subtomogram data analysis approach to study continuous conformational variability of biomolecular complexes, which maps a set of subtomograms into a space of conformations using a reference model and its normal modes. The conformational space permits (i) grouping (and averaging) subtomograms with similar conformations and revealing hidden conformations and (ii) recording animated displacements of the reference model along the densest regions of the space, along trajectories identified by curve fitting of the data in these regions. These HEMNMA-3D outputs could be valuable to cryo-ET studies of molecular mechanisms involved in conformational changes of complexes *in vitro* and *in situ*. HEMNMA-3D is thoroughly tested using synthetic subtomograms and applied to a cryo-ET experimental dataset (nucleosome subtomograms recorded *in situ* in Drosophila interphase nucleus). It provides promising results coherent with previous findings. An open-source software with a graphical user interface is provided for this method with a C++ backend and a Message Passing Interface parallelization scheme.

Both HEMNMA-3D and NMA-based flexible fitting are NMA applications concerned with estimating the molecular conformation in density maps based on an atomic or pseudoatomic reference. However, the purpose of HEMNMA-3D is different from that of the classical NMA-based fitting methods. Classical NMA-based fitting methods aim at determining an atomic representation of an EM density map, which is done by flexible fitting of a given atomic structure into that EM map. The purpose of HEMNMA-3D is to get a low-dimensional representation of the heterogeneity of a given set of EM maps, such as subtomograms. Such low-dimensional representations do not require pushing the limits of the fitting accuracy as in the case of classical flexible fitting of atomic structures into EM maps, which also prevents overfitting. Besides, HEMNMA-3D performs a rigid-body alignment simultaneously with the flexible alignment, which accounts for the missing wedge of the low-SNR subtomograms, whereas classical NMA-based fitting methods typically use high-SNR average consensus EM maps reconstructed from single particle cryo-EM images without missing wedge.

The uniform random distribution of conformations was used in our experiments with synthetic data to show that HEMNMA-3D finds the correct values (within an acceptable error) for any conformation, rotation and translation, and that it does not yield wrong biased solutions (e.g., systematic alignment errors, such as a wrong biased alignment to one or the other conformation and systematic rotational or translational errors). Taking into account the independent analysis of each individual subtomogram, HEMNMA-3D should be able to recover any other conformational distribution, with similar errors to those obtained with the conformational distributions used in this article.

The tests with synthetic data using a pseudoatomic structure from a simulated density map as a reference were used to demonstrate the ability of the method to retrieve the ground truth conformations with a comparable accuracy to the accuracy achieved using an atomic reference despite that (i) the pseudoatomic reference was not used to synthesize the data (the datasets were synthesized using the atomic reference), (ii) pseudoatomic coordinates unlikely coincide with atomic coordinates (pseudoatomic coordinates are obtained through volume-to-pseudoatoms conversion, which does not use any prior information about atoms), and (iii) the method for calculating normal modes is different for a pseudoatomic reference (Cartesian method) and an atomic reference (RTB method). In experimental cases, one could obtain pseudoatoms from a density map of higher resolution than the data itself, if such density map is already available (e.g., an EMDB map obtained by high-resolution single particle cryo-EM reconstruction) or if it can be simulated from an available atomic structure (as in our synthetic data experiments). However, a preferred choice for the reference density map should be a density map from the data itself, which can be obtained by classical subtomogram averaging (without taking into account conformational heterogeneity), as was the case in the nucleosome experiments shown in this article.

The synthetic subtomogram datasets used in this work do not account for crowded molecular environments, radiation dose accumulation during tilting, and differences in CTF defocus over the tilted planes. Nevertheless, the synthesized subtomograms used here were challenging, as containing a small number of voxels and as being obtained by 3D reconstruction from synthetic tilt images affected by strong noise and CTF, which altogether lowered the resolution of the reconstructed subtomograms. The subtomograms were additionally affected by the missing wedge artifacts. Despite such difficult conditions, HEMNMA-3D finds the correct conformational, orientational, and translational parameters, which suggests that it can be useful in practice, and this usefulness was here demonstrated with experimental nucleosome subtomograms.

In experimental cases, such as the nucleosome study shown in this article, the number of used normal modes will always be smaller than the actual number of normal modes (the entire set of modes is too large to be included in our calculations as this would require too long computing times). Therefore, the conformational landscape will always be an approximation of the actual conformational landscape. Small sets of selected potentially relevant modes have been shown to produce good approximation of the actual conformational landscape of the nucleosome studied here by HEMNMA-3D as well as of other complexes studied by HEMNMA. In some cases, a single normal mode could be enough, such as in the case of 70S ribosomes, where the normal mode describing the rotation between the two subunits 30S and 50S was used to analyze conformational and compositional variability of EF-G bound and unbound 70S ribosome cryo-EM dataset (Jin et al., 2014). HEMNMA-3D uses the same software for NMA and the same numerical optimizer (to estimate the amplitudes of normal modes iteratively) as HEMNMA, and it should thus have similar performance as HEMNMA regarding the determination of the conformational landscape using a smaller number of normal modes. HEMNMA-3D software (ConinuousFlex plugin of Scipion V3.0) helps the user decide which normal modes to select, based on the lowest-frequency highest-collectivity criterion and including or not a prior knowledge about the conformational transitions, as is the case with HEMNMA software (Harastani et al., 2020).

HEMNMA-3D can deal with larger sets of subtomograms than those shown in this article. Each subtomogram is analyzed independently of other subtomograms, meaning on a separate computing thread. The same computing time per subtomogram is required for larger and smaller datasets of the same molecular complex and the time required to process the entire dataset varies with the size of the dataset.

## Data Availability Statement

The original contributions presented in the study are included in the article/Supplementary Files. HEMNMA-3D software code is publicly available on Github (https://github.com/scipion-em/scipion-em-continuousflex.git) and is also part of the open-source ContinuousFlex plugin of Scipion V3.0. The nucleosome data used in this article have been deposited in EMPIAR and EMDB databases under the accession codes EMPIAR-10679 and EMD-12699, respectively. Further inquiries can be directed to the corresponding author.

## Author Contributions

MH and SJ designed the method and the experiments with synthetic data. ME obtained the nucleosome subtomograms and their initial average used as the reference for the method in nucleosome experiments. MH implemented the method and performed all the experiments, under the supervision of SJ, and wrote the first draft of the article manuscript, which was finalized by SJ with input from all authors. ME and AL participated in the results interpretation. All authors designed the experiments with nucleosome data, contributed to the manuscript preparation, read, and approved the final version of the manuscript.

## Conflict of Interest

The authors declare that the research was conducted in the absence of any commercial or financial relationships that could be construed as a potential conflict of interest.

## References

[B1] Abeyrathne P. D. San Koh C. Grant T. Grigorieff N. Korostelev A. A. (2016). Ensemble cryo-em uncovers inchworm-like translocation of a viral ires through the ribosome. Elife 5:e14874. 10.7554/eLife.14874 27159452 PMC4896748

[B2] Albert S. Schaffer M. Beck F. Mosalaganti S. Asano S. Thomas H. F.. (2017). Proteasomes tether to two distinct sites at the nuclear pore complex. Proc. Natl. Acad. Sci. U.S.A. 114, 13726–13731. 10.1073/pnas.1716305114 29229809 PMC5748218

[B3] Andén J. Singer A. (2018). Structural variability from noisy tomographic projections. SIAM J. Imaging Sci. 11, 1441–1492. 10.1137/17M1153509 30555617 PMC6294454

[B4] Banerjee S. Bartesaghi A. Merk A. Rao P. Bulfer S. L. Yan Y.. (2016). 2.3 å resolution cryo-em structure of human p97 and mechanism of allosteric inhibition. Science 351, 871–875. 10.1126/science.aad7974 26822609 PMC6946184

[B5] Berghen F. V. Bersini H. (2005). Condor, a new parallel, constrained extension of Powell's Uobyqa algorithm: experimental results and comparison with the dfo algorithm. J. Comput. Appl. Math. 181, 157–175. 10.1016/j.cam.2004.11.029

[B6] Bharat T. A. Scheres S. H. (2016). Resolving macromolecular structures from electron cryo-tomography data using subtomogram averaging in relion. Nat. Protoc. 11, 2054–2065. 10.1038/nprot.2016.124 27685097 PMC5215819

[B7] Böck D. Medeiros J. M. Tsao H. F. Penz T. Weiss G. L. Aistleitner K.. (2017). *In situ* architecture, function, and evolution of a contractile injection system. Science 357, 713–717. 10.1126/science.aan7904 28818949 PMC6485382

[B8] Brüschweiler R. (1995). Collective protein dynamics and nuclear spin relaxation. J. Chem. Phys. 102, 3396–3403. 10.1063/1.469213

[B9] Bykov Y. S. Schaffer M. Dodonova S. O. Albert S. Plitzko J. M. Baumeister W.. (2017). The structure of the copi coat determined within the cell. Elife 6:e32493. 10.7554/eLife.32493 29148969 PMC5716667

[B10] Castaño-Díez D. Zanetti G. (2019). In situ structure determination by subtomogram averaging. Curr. Opin. Struct. Biol. 58, 68–75. 10.1016/j.sbi.2019.05.011 31233977 PMC7116126

[B11] Chen Y. Pfeffer S. Fernández J. J. Sorzano C. O. S. Förster F. (2014). Autofocused 3D classification of cryoelectron subtomograms. Structure 22, 1528–1537. 10.1016/j.str.2014.08.007 25242455

[B12] Chen Y. Pfeffer S. Hrabe T. Schuller J. M. Förster F. (2013). Fast and accurate reference-free alignment of subtomograms. J. Struct. Biol. 182, 235–245. 10.1016/j.jsb.2013.03.002 23523719

[B13] Dashti A. Schwander P. Langlois R. Fung R. Li W. Hosseinizadeh A.. (2014). Trajectories of the ribosome as a brownian nanomachine. Proc. Natl. Acad. Sci. U.S.A. 111, 17492–17497. 10.1073/pnas.1419276111 25422471 PMC4267381

[B14] Davies K. M. Blum T. B. Kühlbrandt W. (2018). Conserved *in situ* arrangement of complex i and iii2 in mitochondrial respiratory chain supercomplexes of mammals, yeast, and plants. Proc. Natl. Acad. Sci. U.S.A. 115, 3024–3029. 10.1073/pnas.1720702115 29519876 PMC5866595

[B15] De la Rosa-Trevín J. Quintana A. Del Cano L. Zaldivar A. Foche I. Gutierrez J.. (2016). Scipion: a software framework toward integration, reproducibility and validation in 3D electron microscopy. J. Struct. Biol. 195, 93–99. 10.1016/j.jsb.2016.04.010 27108186

[B16] Delarue M. Dumas P. (2004). On the use of low-frequency normal modes to enforce collective movements in refining macromolecular structural models. Proc. Natl. Acad. Sci. U.S.A. 101, 6957–6962. 10.1073/pnas.0400301101 15096585 PMC406448

[B17] Durand P. Trinquier G. Sanejouand Y. H. (1994). A new approach for determining low-frequency normal modes in macromolecules. Biopolymers 34, 759–771. 10.1002/bip.360340608

[B18] Elad N. Clare D. K. Saibil H. R. Orlova E. V. (2008). Detection and separation of heterogeneity in molecular complexes by statistical analysis of their two-dimensional projections. J. Struct. Biol. 162, 108–120. 10.1016/j.jsb.2007.11.007 18166488

[B19] Eltsov M. Grewe D. Lemercier N. Frangakis A. Livolant F. Leforestier A. (2018). Nucleosome conformational variability in solution and in interphase nuclei evidenced by cryo-electron microscopy of vitreous sections. Nucleic Acids Res. 46, 9189–9200. 10.1093/nar/gky670 30053160 PMC6158616

[B20] Förster F. Pruggnaller S. Seybert A. Frangakis A. S. (2008). Classification of cryo-electron sub-tomograms using constrained correlation. J. Struct. Biol. 161, 276–286. 10.1016/j.jsb.2007.07.006 17720536

[B21] Frank J. Ourmazd A. (2016). Continuous changes in structure mapped by manifold embedding of single-particle data in cryo-em. Methods 100, 61–67. 10.1016/j.ymeth.2016.02.007 26884261 PMC4848141

[B22] Fu J. Gao H. Frank J. (2007). Unsupervised classification of single particles by cluster tracking in multi-dimensional space. J. Struct. Biol. 157, 226–239. 10.1016/j.jsb.2006.06.012 16931050

[B23] Galaz-Montoya J. G. Flanagan J. Schmid M. F. Ludtke S. J. (2015). Single particle tomography in EMAN2. J. Struct. Biol. 190, 279–290. 10.1016/j.jsb.2015.04.016 25956334 PMC4457691

[B24] Guo Q. Lehmer C. Martínez-Sánchez A. Rudack T. Beck F. Hartmann H.. (2018). *In situ* structure of neuronal C9orf72 Poly-GA aggregates reveals proteasome recruitment. Cell 172, 696–705. 10.1016/j.cell.2017.12.030 29398115 PMC6035389

[B25] Harastani M. Sorzano C. O. S. Jonić S. (2020). Hybrid electron microscopy normal mode analysis with scipion. Prot. Sci. 29, 223–236. 10.1002/pro.3772 31693263 PMC6933837

[B26] Haselbach D. Komarov I. Agafonov D. E. Hartmuth K. Graf B. Dybkov O.. (2018). Structure and conformational dynamics of the human spliceosomal bact complex. Cell 172, 454–464.e11. 10.1016/j.cell.2018.01.010 29361316

[B27] Himes B. A. Zhang P. (2018). emclarity: software for high-resolution cryo-electron tomography and subtomogram averaging. Nat. Methods 15, 955–961. 10.1038/s41592-018-0167-z 30349041 PMC6281437

[B28] Hutchings J. Stancheva V. Miller E. A. Zanetti G. (2018). Subtomogram averaging of copii assemblies reveals how coat organization dictates membrane shape. Nat. Commun. 9:4154. 10.1038/s41467-018-06577-4 30297805 PMC6175875

[B29] Iwasaki W. Miya Y. Horikoshi N. Osakabe A. Taguchi H. Tachiwana H.. (2013). Contribution of histone N-terminal tails to the structure and stability of nucleosomes. FEBS Open Bio 3, 363–369. 10.1016/j.fob.2013.08.007 24251097 PMC3821030

[B30] Jin Q. Sorzano C. O. S. De La Rosa-Trevín J. M. Bilbao-Castro J. R. Núñez-Ramírez R. Llorca O.. (2014). Iterative elastic 3D-to-2D alignment method using normal modes for studying structural dynamics of large macromolecular complexes. Structure 22, 496–506. 10.1016/j.str.2014.01.004 24508340

[B31] Jonić S. (2017). Computational methods for analyzing conformational variability of macromolecular complexes from cryo-electron microscopy images. Curr. Opin. Struct. Biol. 43, 114–121. 10.1016/j.sbi.2016.12.011 28088125

[B32] Jonić S. Sorzano C. (2016). Coarse-graining of volumes for modeling of structure and dynamics in electron microscopy: algorithm to automatically control accuracy of approximation. IEEE J. Select. Top. Signal Process. 10, 161–173. 10.1109/JSTSP.2015.2489186

[B33] Kaplan M. Ghosal D. Subramanian P. Oikonomou C. M. Kjaer A. Pirbadian S.. (2019). The presence and absence of periplasmic rings in bacterial flagellar motors correlates with stator type. Elife 8:e43487. 10.7554/eLife.43487 30648971 PMC6375700

[B34] Katsevich E. Katsevich A. Singer A. (2015). Covariance matrix estimation for the cryo-em heterogeneity problem. SIAM J. Imaging Sci. 8, 126–185. 10.1137/130935434 25699132 PMC4331039

[B35] Kovacs J. A. Chacón P. Cong Y. Metwally E. Wriggers W. (2003). Fast rotational matching of rigid bodies by fast Fourier transform acceleration of five degrees of freedom. Acta Crystallogr. D 59, 1371–1376. 10.1107/S0907444903011247 12876338

[B36] Kovacs J. A. Wriggers W. (2002). Fast rotational matching. Acta Crystallogr. D Biol. Crystallogr. 58, 1282–1286. 10.1107/S0907444902009794 12136139

[B37] Kovtun O. Leneva N. Bykov Y. S. Ariotti N. Teasdale R. D. Schaffer M.. (2018). Structure of the membrane-assembled retromer coat determined by cryo-electron tomography. Nature 561, 561–564. 10.1038/s41586-018-0526-z 30224749 PMC6173284

[B38] Kufareva I. Abagyan R. (2011). “Methods of protein structure comparison,” in Homology Modeling, Orry A. J. W. Abagyan R. (New York, NY; Heidelberg; Dordrecht; London: Springer), 231–257. 10.1007/978-1-61779-588-6_10 PMC4321859 22323224

[B39] Leigh K. E. Navarro P. P. Scaramuzza S. Chen W. Zhang Y. Castaño-Díez D.. (2019). Subtomogram averaging from cryo-electron tomograms. Methods Cell Biol. 152, 217–259. 10.1016/bs.mcb.2019.04.003 31326022

[B40] Lyumkis D. Brilot A. F. Theobald D. L. Grigorieff N. (2013). Likelihood-based classification of cryo-em images using frealign. J. Struct. Biol. 183, 377–388. 10.1016/j.jsb.2013.07.005 23872434 PMC3824613

[B41] Ma J. (2005). Usefulness and limitations of normal mode analysis in modeling dynamics of biomolecular complexes. Structure 13, 373–380. 10.1016/j.str.2005.02.002 15766538

[B42] Mahalanobis P. C. (1936). On the Generalized Distance in Statistics. Kolkata: National Institute of Science of India.

[B43] Mahamid J. Pfeffer S. Schaffer M. Villa E. Danev R. Cuellar L. K.. (2016). Visualizing the molecular sociology at the hela cell nuclear periphery. Science 351, 969–972. 10.1126/science.aad8857 26917770

[B44] Mattei S. Glass B. Hagen W. J. H. Kräusslich H. G. Briggs J. A. G. (2016). The structure and flexibility of conical HIV-1 capsids determined within intact virions. Science 354, 1434–1437. 10.1126/science.aah4972 27980210

[B45] Moebel E. Kervrann C. (2020). A monte carlo framework for missing wedge restoration and noise removal in cryo-electron tomography. J. Struct. Biol. X 4:100013. 10.1016/j.yjsbx.2019.100013 32647817 PMC7337055

[B46] Mosalaganti S. Kosinski J. Albert S. Schaffer M. Strenkert D. Salomé P. A.. (2018). *In situ* architecture of the algal nuclear pore complex. Nat. Commun. 9:2361. 10.1038/s41467-018-04739-y 29915221 PMC6006428

[B47] Müller C. Schlauderer G. Reinstein J. Schulz G. E. (1996). Adenylate kinase motions during catalysis: an energetic counterweight balancing substrate binding. Structure 4, 147–156. 10.1016/S0969-2126(96)00018-4 8805521

[B48] Nogales-Cadenas R. Jonic S. Tama F. Arteni A. Tabas-Madrid D. Vázquez M.. (2013). 3DEM loupe: analysis of macromolecular dynamics using structures from electron microscopy. Nucleic Acids Res. 41, W363–W367. 10.1093/nar/gkt385 23671335 PMC3692114

[B49] Park D. Lara-Tejero M. Waxham M. N. Li W. Hu B. Galán J. E.. (2018). Visualization of the type III secretion mediated salmonella-host cell interface using cryo-electron tomography. Elife 7:e39514. 10.7554/eLife.39514 30281019 PMC6175578

[B50] Penczek P. A. Frank J. Spahn C. M. (2006). A method of focused classification, based on the bootstrap 3D variance analysis, and its application to EF-G-dependent translocation. J. Struct. Biol. 154, 184–194. 10.1016/j.jsb.2005.12.013 16520062

[B51] Penczek P. A. Kimmel M. Spahn C. M. (2011). Identifying conformational states of macromolecules by eigen-analysis of resampled cryo-em images. Structure 19, 1582–1590. 10.1016/j.str.2011.10.003 22078558 PMC3255080

[B52] Peng L. M. Ren G. Dudarev S. Whelan M. (1996). Robust parameterization of elastic and absorptive electron atomic scattering factors. Acta Crystallogr. A Foundat. Crystallogr. 52, 257–276. 10.1107/S0108767395014371

[B53] Pfeffer S. Dudek J. Schaffer M. Ng B. G. Albert S. Plitzko J. M.. (2017). Dissecting the molecular organization of the translocon-associated protein complex. Nat. Commun. 8:14516. 10.1038/ncomms14516 28218252 PMC5321747

[B54] Rapisarda C. Cherrak Y. Kooger R. Schmidt V. Pellarin R. Logger L.. (2019). *In situ* and high-resolution cryo-em structure of a bacterial type VI secretion system membrane complex. EMBO J. 38:e100886. 10.15252/embj.2018100886 30877094 PMC6517824

[B55] Riedel C. Vasishtan D. Siebert C. A. Whittle C. Lehmann M. J. Mothes W.. (2017). Native structure of a retroviral envelope protein and its conformational change upon interaction with the target cell. J. Struct. Biol. 197, 172–180. 10.1016/j.jsb.2016.06.017 27345930 PMC5182179

[B56] Scheres S. H. (2012). Relion: implementation of a bayesian approach to cryo-em structure determination. J. Struct. Biol. 180, 519–530. 10.1016/j.jsb.2012.09.006 23000701 PMC3690530

[B57] Scheres S. H. Melero R. Valle M. Carazo J. M. (2009). Averaging of electron subtomograms and random conical tilt reconstructions through likelihood optimization. Structure 17, 1563–1572. 10.1016/j.str.2009.10.009 20004160 PMC2940245

[B58] Schur F. K. Obr M. Hagen W. J. Wan W. Jakobi A. J. Kirkpatrick J. M.. (2016). An atomic model of HIV-1 Capsid-SP1 reveals structures regulating assembly and maturation. Science 353, 506–508. 10.1126/science.aaf9620 27417497

[B59] Sorzano C. Jonic S. Núñez-Ramírez R. Boisset N. Carazo J. (2007). Fast, robust, and accurate determination of transmission electron microscopy contrast transfer function. J. Struct. Biol. 160, 249–262. 10.1016/j.jsb.2007.08.013 17911028

[B60] Sorzano C. O. de la Rosa Trevín J. Otón J. Vega J. Cuenca J. Zaldívar-Peraza A.. (2013). “Semiautomatic, high-throughput, high-resolution protocol for three-dimensional reconstruction of single particles in electron microscopy,” in Nanoimaging, Sousa A. A. Kruhlak M. J. (New York, NY; Heidelberg; Dordrecht; London: Springer), 171–193. 10.1007/978-1-62703-137-0_11 23086876

[B61] Sorzano C. O. S. Alvarez-Cabrera A. L. Kazemi M. Carazo J. M. Jonić S. (2016). Structmap: elastic distance analysis of electron microscopy maps for studying conformational changes. Biophys. J. 110, 1753–1765. 10.1016/j.bpj.2016.03.019 27119636 PMC4850348

[B62] Sorzano C. O. S. de La Rosa-Trevín J. M. Tama F. Jonić S. (2014). Hybrid electron microscopy normal mode analysis graphical interface and protocol. J. Struct. Biol. 188, 134–141. 10.1016/j.jsb.2014.09.005 25268657

[B63] Sorzano C. O. S. Jiménez A. Mota J. Vilas J. L. Maluenda D. Martínez M.. (2019). Survey of the analysis of continuous conformational variability of biological macromolecules by electron microscopy. Acta Crystallogr. F 75, 19–32. 10.1107/S2053230X18015108 30605122 PMC6317454

[B64] Stölken M. Beck F. Haller T. Hegerl R. Gutsche I. Carazo J. M.. (2011). Maximum likelihood based classification of electron tomographic data. J. Struct. Biol. 173, 77–85. 10.1016/j.jsb.2010.08.005 20719249

[B65] Suhre K. Navaza J. Sanejouand Y. H. (2006). Norma: a tool for flexible fitting of high-resolution protein structures into low-resolution electron-microscopy-derived density maps. Acta Crystallogr. D Biol. Crystallogr. 62, 1098–1100. 10.1107/S090744490602244X 16929111

[B66] Suhre K. Sanejouand Y. H. (2004). ELNEMO: a normal mode web server for protein movement analysis and the generation of templates for molecular replacement. Nucleic Acids Res. 32, W610–W614. 10.1093/nar/gkh368 15215461 PMC441506

[B67] Tagare H. D. Kucukelbir A. Sigworth F. J. Wang H. Rao M. (2015). Directly reconstructing principal components of heterogeneous particles from cryo-em images. J. Struct. Biol. 191, 245–262. 10.1016/j.jsb.2015.05.007 26049077 PMC4536832

[B68] Tama F. Brooks C. L. III. (2006). Symmetry, form, and shape: guiding principles for robustness in macromolecular machines. Annu. Rev. Biophys. Biomol. Struct. 35, 115–133. 10.1146/annurev.biophys.35.040405.102010 16689630

[B69] Tama F. Gadea F. X. Marques O. Sanejouand Y. H. (2000). Building-block approach for determining low-frequency normal modes of macromolecules. Proteins 41, 1–7. 10.1002/1097-0134(20001001)41:1<1::AID-PROT10>3.0.CO;2-P 10944387

[B70] Tama F. Miyashita O. Brooks III C. L. (2004b). Normal mode based flexible fitting of high-resolution structure into low-resolution experimental data from cryo-em. J. Struct. Biol. 147, 315–326. 10.1016/j.jsb.2004.03.002 15450300

[B71] Tama F. Miyashita O. Brooks C. L. (2004a). Flexible multi-scale fitting of atomic structures into low-resolution electron density maps with elastic network normal mode analysis. J. Mol. Biol. 337, 985–999. 10.1016/j.jmb.2004.01.048 15033365

[B72] Tama F. Sanejouand Y. H. (2001). Conformational change of proteins arising from normal mode calculations. Prot. Eng. 14, 1–6. 10.1093/protein/14.1.1 11287673

[B73] Tama F. Wriggers W. Brooks C. L. III. (2002). Exploring global distortions of biological macromolecules and assemblies from low-resolution structural information and elastic network theory. J. Mol. Biol. 321, 297–305. 10.1016/S0022-2836(02)00627-7 12144786

[B74] Tirion M. M. (1996). Large amplitude elastic motions in proteins from a single-parameter, atomic analysis. Phys. Rev. Lett. 77, 1905–1908. 10.1103/PhysRevLett.77.1905 10063201

[B75] Wan W. Briggs J. (2016). “Chapter 13–cryo-electron tomography and subtomogram averaging,” in The Resolution Revolution: Recent Advances in cryoEM, Volume 579 of Methods in Enzymology, Crowther R. (Cambridge: Academic Press), 329–367. 10.1016/bs.mie.2016.04.014 27572733

[B76] Wan W. Kolesnikova L. Clarke M. Koehler A. Noda T. Becker S.. (2017). Structure and assembly of the ebola virus nucleocapsid. Nature 551, 394–397. 10.1038/nature24490 29144446 PMC5714281

[B77] Wang Y. Rader A. Bahar I. Jernigan R. L. (2004). Global ribosome motions revealed with elastic network model. J. Struct. Biol. 147, 302–314. 10.1016/j.jsb.2004.01.005 15450299

[B78] Xu M. Beck M. Alber F. (2012). High-throughput subtomogram alignment and classification by fourier space constrained fast volumetric matching. J. Struct. Biol. 178, 152–164. 10.1016/j.jsb.2012.02.014 22420977 PMC3821800

[B79] Zhai X. Lei D. Zhang M. Liu J. Wu H. Yu Y.. (2020). LoTTor: an algorithm for missing-wedge correction of the low-tilt tomographic 3D reconstruction of a single-molecule structure. Sci. Rep. 10:10489. 10.1038/s41598-020-66793-1 32591588 PMC7320192

[B80] Zhang L. Ren G. (2012). High-resolution single-molecule structure revealed by electron microscopy and individual particle electron tomography. J. Phys. Chem. Biophys. 2:2. 10.4172/2161-0398.1000e103 PMC10538445 37772199

[B81] Zhou A. Rohou A. Schep D. G. Bason J. V. Montgomery M. G. Walker J. E.. (2015). Structure and conformational states of the bovine mitochondrial atp synthase by cryo-em. Elife 4:e10180. 10.7554/eLife.10180 26439008 PMC4718723

[B82] Zlatanova J. Bishop T. C. Victor J. Jackson V. van Holde K. V. (2009). The nucleosome family: dynamic and growing. Structure 17, 160–171. 10.1016/j.str.2008.12.016 19217387

