# Supplementary material for: HEMNMA-3D: Cryo Electron Tomography Method Based on Normal Mode Analysis to Study Continuous Conformational Variability of Macromolecular Complexes
Source: Front Mol Biosci. 2021 May 19;8:663121. doi: 10.3389/fmolb.2021.663121 (PMC8170028; doi:10.3389/fmolb.2021.663121)
Supplement: Supplementary file 1 [file Data_Sheet_1.pdf]

Supplementary Material for HEMNMA-3D: Cryo Electron Tomography Method Based on Normal Mode Analysis to Study Continuous Conformational Variability of Macromolecular Complexes

Mohamad Harastani [1] **Mikhail Eltsov** [2] **Amélie Leforestier** [3] **Slavica Jonic** [1,*]

[1] *IMPMC - UMR 7590 CNRS, Sorbonne Université, Muséum National d'Histoire Naturelle, Paris, France*
[2] *Department of Integrated Structural Biology, Institute of Genetics and Molecular and Cellular Biology, Illkirch, France*
[3] *Laboratoire de Physique des Solides (LPS), UMR 8502 CNRS, Université Paris-Saclay, Orsay, France*

Correspondence*:
Slavica Jonic, IMPMC - UMR CNRS 7590, Sorbonne Univerisité, 4 Place Jussieu, 75005 Paris, France
slavica.jonic@upmc.fr

1 NUCLEOSOME DATA PREPARATION AND ACQUISITION

The Drosophila embryo cryo-sample preparation, vitreous sectioning, tilt series acquisition and tomogram reconstruction were performed as described in Eltsov et al. (2018). Briefly, Drosophila embryos (Bloomington Stock number 30564) were frozen by high-pressure freezing using an HPM 010 machine (ABRA Fluid AG). The vitreous sections were obtained using a 25° diamond knife (Diatome) and Leica FC6/UC6 ultramicrotome (Leica Microsystems). Sections were collected on C-flat CF-2/1 grids (Electron Microscopy Sciences) and transferred into a Titan Krios (FEI, Thermofischer, Eindhoven, The Netherlands) operated at 300 kV equipped with a GATAN GIF Quantum SE post-column energy filter and K2 Summit direct electron detector (Gatan, Pleasanton, USA). Tilt series were recorded using Serial EM software (Mastronarde (2005)) at a nominal magnification of 64000 × (2.2 Å/pixel), and a target defocus of -3.5 μm. The dose-symmetric recording scheme (Hagen et al. (2017)) was applied within an angular range from 60° to +60°, with a starting angle 0° and an angular increment of 2°. The electron dose was set to 1.5 e^-/ Å^2 for individual tilt images, corresponding to the total dose of 91.5 e^-/ Å^2 for the complete tilt series. A marker-less tilt series alignment was done in IMOD (Kremer et al. (1996)), three dimensional CTF correction and weighted backprojection with the voxel size of 4.4 Å were performed using EmSART script (Kunz and Frangakis (2014, 2017)) provided by Achilleas Frangakis. A slice of the experimental nucleosome tomographic data is shown in Figure S1. The reconstructed volumes were denoised using 3D non-linear anisotropic diffusion filter of Etomo of IMOD (k = 1, 15 iterations). Nucleosomes were manually picked in IMOD. Then, 64^3 voxel subtomograms (voxel size of 4.4 Å) were extracted from the original non-denoised volumes. To refine manually-picked nucleosome coordinates, subtomogram alignment and averaging were performed with SubTomogramAveraging script using a sum of the randomly rotated subtomograms as an initial reference. Alignment of subtomograms was performed in two steps. Initially, a

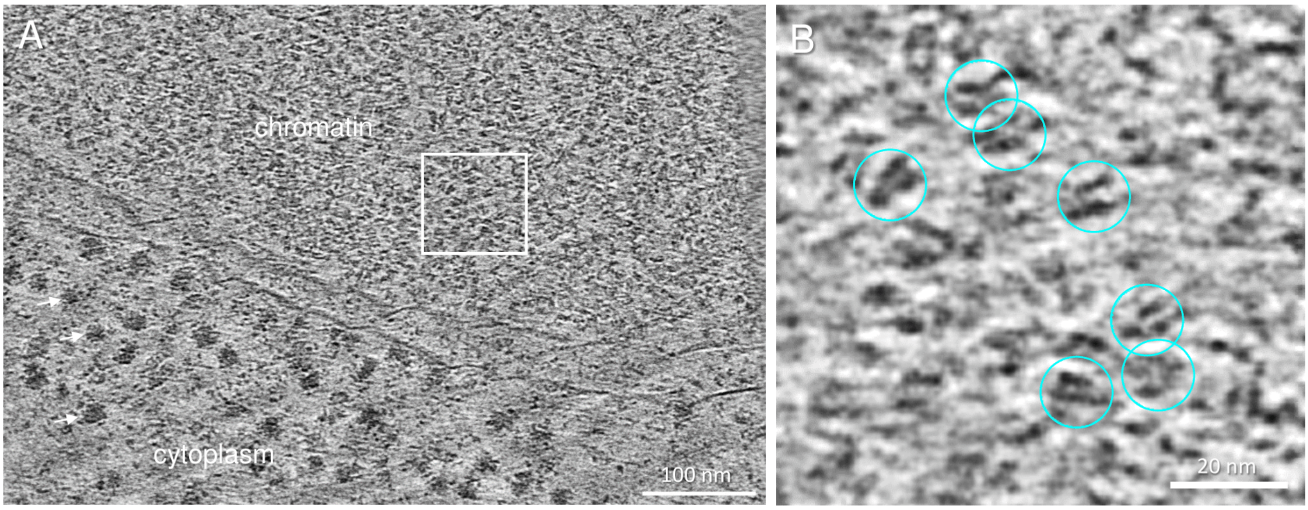

Figure S1. A slice of the experimental nucleosome tomographic data: (A) A 5-nm thick slice through a tomographic reconstruction showing an area of compact chromatin at a nuclear periphery (chromatin) that is easily distinguished from cytoplasm filled with ribosomes (arrows). (B) An enlargement of the chromatin area outlined with a white square in (A). Circles indicate positions of nucleosomes selected for subtomogram extraction.

bandpass filter was applied with a low cutoff frequency of 3 reciprocal-space pixels ($\approx$ 1/94 Å^{-1}), a high cutoff frequency of 8 reciprocal-space pixels ($\approx$ 1/35 Å^{-1}), and a Gaussian edge smoothing with a standard deviation of 3 reciprocal-space pixels. Ten iterations of an unconstrained rotational search (three rotational degrees of freedom) were performed with an angular sampling step of 10°, and a translational search (three translational degrees of freedom) was performed within a radius of 5 real-space pixels (22 Å). In the second step, a bandpass filter was applied with low and high cutoff frequencies of 3 reciprocal-space pixels ($\approx$ 1/94 Å^{-1}) and 15 reciprocal-space pixels ($\approx$ 1/19 Å^{-1}), respectively, and a Gaussian edge smoothing with a standard deviation of 3 reciprocal-space pixels. At that step, 20 iterations of the rotational search were performed with an angular sampling step of 2°, constrained to 20° around the orientation found in the previous step, and the translational search radius was reduced to 3 real-space pixels (13.2 Å). The cross-correlation between the last several iteration averages ($\approx$ 0.994) indicated the stabilization of the subtomogram alignment. A new set of subtomograms of the same dimensions and voxel size was extracted at the refined nucleosome positions and exported into HEMNMA-3D. The nucleosome data used in this article have been deposited in EMPIAR and EMDB databases under the accession codes EMPIAR-10679 and EMD-12699, respectively.

2 ADDITIONAL SYNTHETIC DATA TESTS WITH DIFFERENT NOISE LEVELS

Additional tests were performed on HEMNMA-3D using synthesized datasets at different noise levels of conformationally heterogeneous subtomograms that mimic continuous conformational variability. The noise levels were chosen as A) without noise, B) SNR = 0.4, C) SNR = 0.1, D) SNR = 0.04, E) SNR = 0.01 and F) SNR = 0.005. Each dataset comprises 200 synthetic subtomograms representing a continuum of conformations of the same PDB:4AKE structure. The flowchart for the data generation procedure is shown in the article manuscript Figure 4. Here, the amplitude value of normal mode 7 is chosen randomly in the range {-300, 300} and the amplitude value mode 8 as half of the value of mode 7. More details on the synthesis of the data will be found in the main article manuscript Section 3.1. The goal in this experiment is to find a solution for the inverse problem of finding the conformation of the structure in each subtomogram

using the combined elastic and rigid-body alignment of a reference model with the subtomograms in the different-in-noise datasets. Figure S2 presents the estimated amplitudes of modes 7 and 8 (the estimated amplitude of mode 9 is close to 0 and is not shown in the plots). Table S1 shows the mean absolute error and the standard deviation between the estimated and ground-truth normal-mode amplitudes.

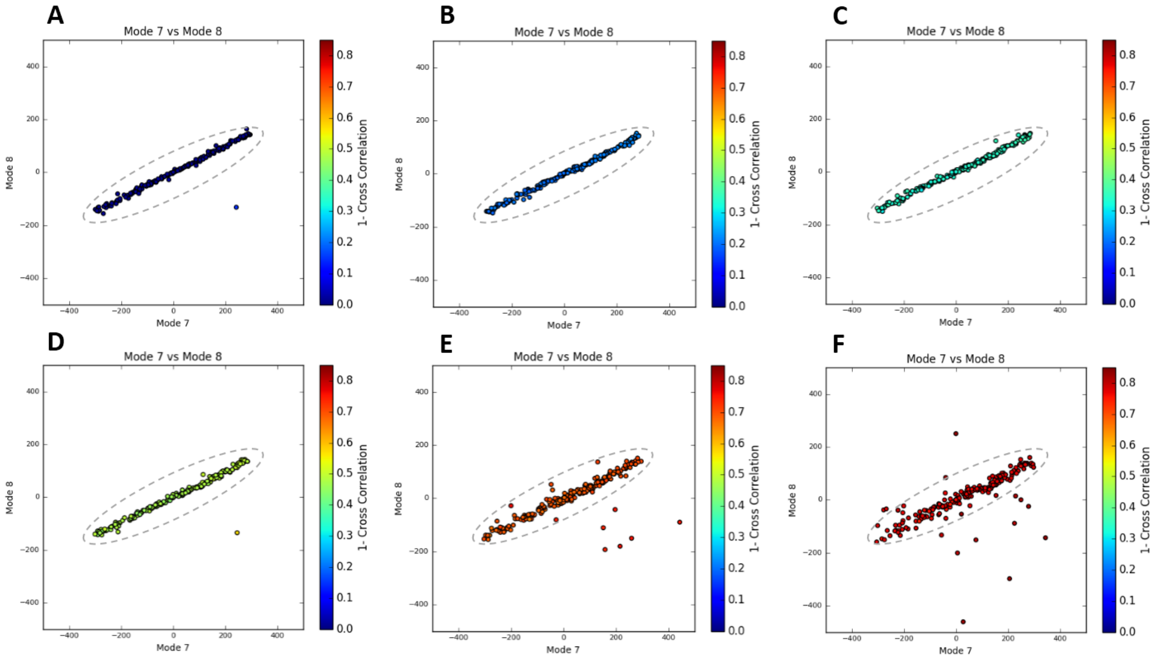

Figure S2. Plots showing the output of the 3D-to-3D elastic and rigid-body alignment module of HEMNMA-3D with synthetic datasets at different noise levels (synthetic subtomograms are simulating continuous conformational heterogeneity), using the atomic structure (chain A of PDB:4AKE) and its normal modes to estimate the conformational parameters (normal-mode amplitudes) and rigid-body parameters (orientation and shift) of the molecules in the input synthetic subtomograms. **(A)** Without noise, **(B)** SNR = 0.4, **(C)** SNR = 0.1, **(D)** SNR = 0.04, **(E)** SNR = 0.01, **(F)** SNR = 0.005. The goal was to retrieve the ground-truth relationship between the amplitudes along normal modes 7 and 8 (ideally linear relationship, with the amplitude of normal modes 8 equals to half the amplitude of mode 7); each point in the plot represents a subtomogram, and close points represent similar conformations. Note that the dashed ellipses contain the data points where the p-value is specified in Table S1.

Noise and CTF		Mode 7		Mode 8		Mode 9		p-value	Samples
**Defocus [µm]**	**SNR**	**mean**	**std**	**mean**	**std**	**mean**	**std**		
No Noise		6.38	4.47	3.37	3.51	3.70	3.85	P $>10^{-9}$	199/200
-1	0.4	8.23	6.35	7.49	5.29	5.64	4.02	P >0	200/200
-1	0.1	8.26	6.34	8.10	5.83	5.85	4.26	P >0	200/200
-1	0.04	11.14	7.20	8.11	5.89	6.98	4.83	P $>10^{-9}$	199/200
-1	0.01	26.68	9.00	13.11	8.75	16.59	9.26	p >0.01	190/200
-1	0.005	35.86	14.20	20.87	11.02	19.40	9.53	p >0.01	187/200

Table S1. Mean absolute error and standard deviation between the estimated and ground-truth normal-mode amplitudes obtained with HEMNMA-3D synthetic datasets for different noise levels, using an atomic structure as an input reference. The corresponding region for the p-value is shown in Figure S2.

3 ADDITIONAL VISUALIZATION ON THE RESULTS WITH *IN SITU* CRYO-ET NUCLEOSOME DATA

Figure S3 represents an overlapping comparison between the atomic nucleosome structure PDB:3w98 and the four group averages obtained from HEMNMA-3D for the nucleosome dataset *in situ*.

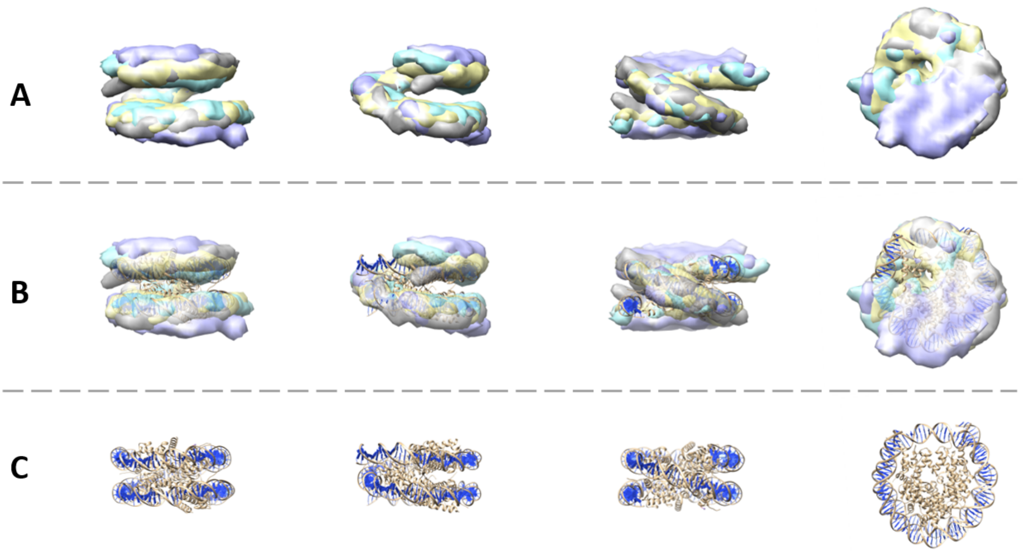

Figure S3. Comparison of the atomic nucleosome structure PDB:3w98 with the four *in situ* nucleosome subtomogram averages obtained with HEMNMA-3D (the experiment shown in Figure 10 in the main article manuscript, which used a preliminary nucleosome subtomogram average as input reference density map for HEMNMA-3D). **(A)** Four views of the four subtomogram averages overlapped. **(B)** Four views of the four averages overlapped at 50% transparency with PDB:3w98. **(C)** Four views of PDB:3w98.

REFERENCES

Eltsov, M., Grewe, D., Lemercier, N., Frangakis, A., Livolant, F., and Leforestier, A. (2018). Nucleosome conformational variability in solution and in interphase nuclei evidenced by cryo-electron microscopy of vitreous sections. *Nucleic acids research* 46, 9189–9200

Hagen, W. J., Wan, W., and Briggs, J. A. (2017). Implementation of a cryo-electron tomography tilt-scheme optimized for high resolution subtomogram averaging. *Journal of structural biology* 197, 191–198

Kremer, J. R., Mastronarde, D. N., and McIntosh, J. R. (1996). Computer visualization of three-dimensional image data using imod. *Journal of structural biology* 116, 71–76

Kunz, M. and Frangakis, A. S. (2014). Super-sampling sart with ordered subsets. *Journal of structural biology* 188, 107–115

Kunz, M. and Frangakis, A. S. (2017). Three-dimensional ctf correction improves the resolution of electron tomograms. *Journal of structural biology* 197, 114–122

Mastronarde, D. N. (2005). Automated electron microscope tomography using robust prediction of specimen movements. *Journal of structural biology* 152, 36–51
